# Selective activations and functional connectivities to the sight of faces, scenes, body parts and tools in visual and non-visual cortical regions leading to the human hippocampus

**DOI:** 10.1007/s00429-024-02811-6

**Published:** 2024-06-05

**Authors:** Edmund T. Rolls, Jianfeng Feng, Ruohan Zhang

**Affiliations:** 1 Department of Computer Science, University of Warwick, Coventry, CV4 7AL UK; 2 Institute of Science and Technology for Brain Inspired Intelligence, Fudan University, Shanghai, 200403 China; 3 Oxford Centre for Computational Neuroscience, Oxford, UK

**Keywords:** Human connectome, Human visual cortex, Human visual pathways, Activations to faces, places, body parts, and tools, Ventromedial visual cortical stream, Cortical scene regions

## Abstract

Connectivity maps are now available for the 360 cortical regions in the Human Connectome Project Multimodal Parcellation atlas. Here we add function to these maps by measuring selective fMRI activations and functional connectivity increases to stationary visual stimuli of faces, scenes, body parts and tools from 956 HCP participants. Faces activate regions in the ventrolateral visual cortical stream (FFC), in the superior temporal sulcus (STS) visual stream for face and head motion; and inferior parietal visual (PGi) and somatosensory (PF) regions. Scenes activate ventromedial visual stream VMV and PHA regions in the parahippocampal scene area; medial (7m) and lateral parietal (PGp) regions; and the reward-related medial orbitofrontal cortex. Body parts activate the inferior temporal cortex object regions (TE1p, TE2p); but also visual motion regions (MT, MST, FST); and the inferior parietal visual (PGi, PGs) and somatosensory (PF) regions; and the unpleasant-related lateral orbitofrontal cortex. Tools activate an intermediate ventral stream area (VMV3, VVC, PHA3); visual motion regions (FST); somatosensory (1, 2); and auditory (A4, A5) cortical regions. The findings add function to cortical connectivity maps; and show how stationary visual stimuli activate other cortical regions related to their associations, including visual motion, somatosensory, auditory, semantic, and orbitofrontal cortex value-related, regions.

**Supplementary Information:**

The online version contains supplementary material available at 10.1007/s00429-024-02811-6.

## Introduction

The aim of this research is to add function by using task-related fMRI using visual stimuli to the maps of cortical connectivity using the Human Connectome Project Multimodal Parcellation atlas HCP-MMP1 (Glasser et al. 2016a) that have been generated using effective and functional connectivity, and diffusion tractography with Human Connectome Project data.

The HCP-MMP1 (Glasser et al. 2016a) is a well-founded parcellation of the human cerebral cortex into 360 cortical regions that utilises evidence from anatomy (cortical thickness and cortical myelin), functional connectivity, and task-related fMRI (Glasser et al. 2016a). This atlas provides a reference system that could be used in many investigations of human cortical function, to provide a reference standard to enable findings from different investigations to be compared. The HCP-MMP1 (Glasser et al. 2016a) has been extended to include 66 subcortical areas (Huang et al. 2022). The HCP-MMP1 is the best cortical atlas we know for delineating the smallest cortical regions that can be reliably identified in humans, which may be building blocks of cortical function and provide a basis for advancing our understanding of cortical function (Rolls 2023c). It contrasts with many earlier parcellations of the cerebral cortex that are less computationally useful as they are based on gross topology (Rolls et al. 2015, 2020), or on cortical regions categorised primarily by functional connectivity (Power et al. 2011).

Maps of cortical connectivity have been generated for many cortical systems using this HCP-MMP1 atlas using effective connectivity, functional connectivity, and diffusion tractography. Effective connectivity measures the connectivity in each direction between each pair of brain regions by using time delays (Rolls et al. 2022b), and was complemented by measurement of functional connectivity, which given that it is based on Pearson correlations, can provide evidence about interactions between brain regions, but not about the direction or causality of effects (Ma et al. 2022; Rolls et al. 2023c). These methods were complemented by diffusion tractography which can measure direct connections between brain regions though not about the direction of connections (Huang et al. 2021; Rolls et al. 2023c). These three types of connectivity maps for the human cerebral cortex have been generated for the visual cortical regions (Rolls et al. 2023b; Rolls et al. 2023a, b, c, d, e, f, g, h; Rolls 2024a); the posterior parietal cortex (Rolls et al. 2023e); the orbitofrontal cortex, anterior cingulate cortex, and ventromedial prefrontal cortex (Rolls et al. 2023c); the posterior cingulate and medial parietal cortex (Rolls et al. 2023b); the auditory cortex (Rolls et al. 2023a); the amygdala compared to the orbitofrontal cortex (Rolls et al. 2023a); the prefrontal and somatosensory cortex (Rolls et al. 2023d); the frontal pole cortex (Rolls et al. 2024a, b); and the hippocampal memory system (Huang et al. 2021; Ma et al. 2022; Rolls et al. 2022b).

These effective and functional connectivity maps of the 360 regions in the HCP-MMP1 atlas were generated with resting state fMRI, which because a task is not being performed may help to provide a foundation for understanding the underlying connectivity of the brain. But it is necessary to link the connectivity maps of the human brain to the functions of each cortical region, in order to understand better the flow of information through the brain, by providing evidence about the functions in which each cortical region is involved.

In the present research, we therefore measured the activations of each of the 360 cortical regions in the HCP-MMP1 atlas in task-related fMRI to different types of visual stimuli, which were stationary views of faces, scenes, body parts, and tools, in data collected for 956 participants by the HCP (Barch et al. 2013; Glasser et al. 2016b) that were analysed here. All the data were in the surface-based version of the HCP-MMP1 atlas, as that provides the most accurate identification of each cortical region (Glasser et al. 2016a). In order to identify cortical regions selectively activated by each of faces, scenes, body parts, and tools, we measured the activations to each of these stimulus types compared to the mean activation averaged across all four types of visual stimuli. To complement the stimulus-selective activations to each of the four visual stimulus types, we also measured the selective functional connectivity between cortical regions using again the functional connectivity map for each stimulus type compared to the mean functional connectivity map across all four visual stimulus types. Although there has been much previous research on human brain activations to faces (Kanwisher et al. 1997; Spiridon et al. 2006; Vul et al. 2012; Weiner and Grill-Spector 2015), scenes (Epstein and Kanwisher 1998; Epstein and Julian 2013; Epstein and Baker 2019; Tsitsiklis et al. 2020), body parts (Pitcher et al. 2011; Vul et al. 2012; Weiner and Grill-Spector 2013; Deen et al. 2015; Orban et al. 2021; Urgen and Orban 2021; Kosakowski et al. 2022; Rolls 2023a, 2024a), tools (Kastner et al. 2017; Maravita and Romano 2018) etc., we note that the aim of the research here is somewhat different, namely to measure the selective activations and increases in functional connectivity using the regions defined in the HCP-MMP1 atlas, partly because this atlas provides a well-founded framework for specifying cortical regions and comparing results between investigations, and importantly to add function to the connectivity maps for the human connectome referred to above. This aim is important for building a framework for better understanding human cerebral cortex function in health and in disease (Rolls 2023c).

New and key aspects of the research described here are as follows. First, we show the selective activations (against a mean baseline) to stationary images of faces, scenes, body parts, and tools provided for all 360 regions in the HCP-MMP atlas with 956 participants. This provides the largest analysis we know of for example scene areas in the human brain. Second, because we analyse the activations present in every HCP-MMP cortical region, we are able to go beyond describing the activation to a class of stimulus by one or several peaks identified by MNI coordinates, we demonstrate the extent to which the cortical activations can in a graded way be found in a number of cortical regions, which moreover can extend beyond classical visual regions to semantically related cortical regions such as somatosensory and auditory and orbitofrontal cortex regions depending on the type of the visual stimulus. Third, we are able to analyse across the whole cortex with 360 cortical regions the selectively high functional connectivities to images of faces, scenes, body parts, and tools provided for all 360 regions in the HCP-MMP atlas with 956 participants, to show how the functional connectivity between different cortical regions changes when the processing is changed by different types of visual stimuli. Fourth, we are able to identify cortical regions and pathways that transmit information beyond primarily visual cortical regions to the hippocampal memory system for different types of stimuli (e.g. scenes vs. faces and objects), which is a topic of great current interest given that the evidence here is on humans, a primate in which the visual representation is of scenes more than of places (Rolls 2023a). Fifth, we are able to show here that even stationary visual stimuli activate visual motion regions of the human cortex depending on the extent to which the stimuli (e.g. tools) imply motion, compared to other visual stimuli (e.g. scenes) that do not. Overall, the analyses described here add all-important function to the effective and functional connectivity and tractography maps for the same cortical regions in the HCP-MMP atlas measured also with HCP data (Huang et al. 2021; Ma et al. 2022; Rolls 2022; Rolls et al. 2022b, 2023b; Rolls et al. 2023c, d, e; Rolls et al. 2023a).

## Methods

### HCP task and working memory paradigm

**Fig. 1:**
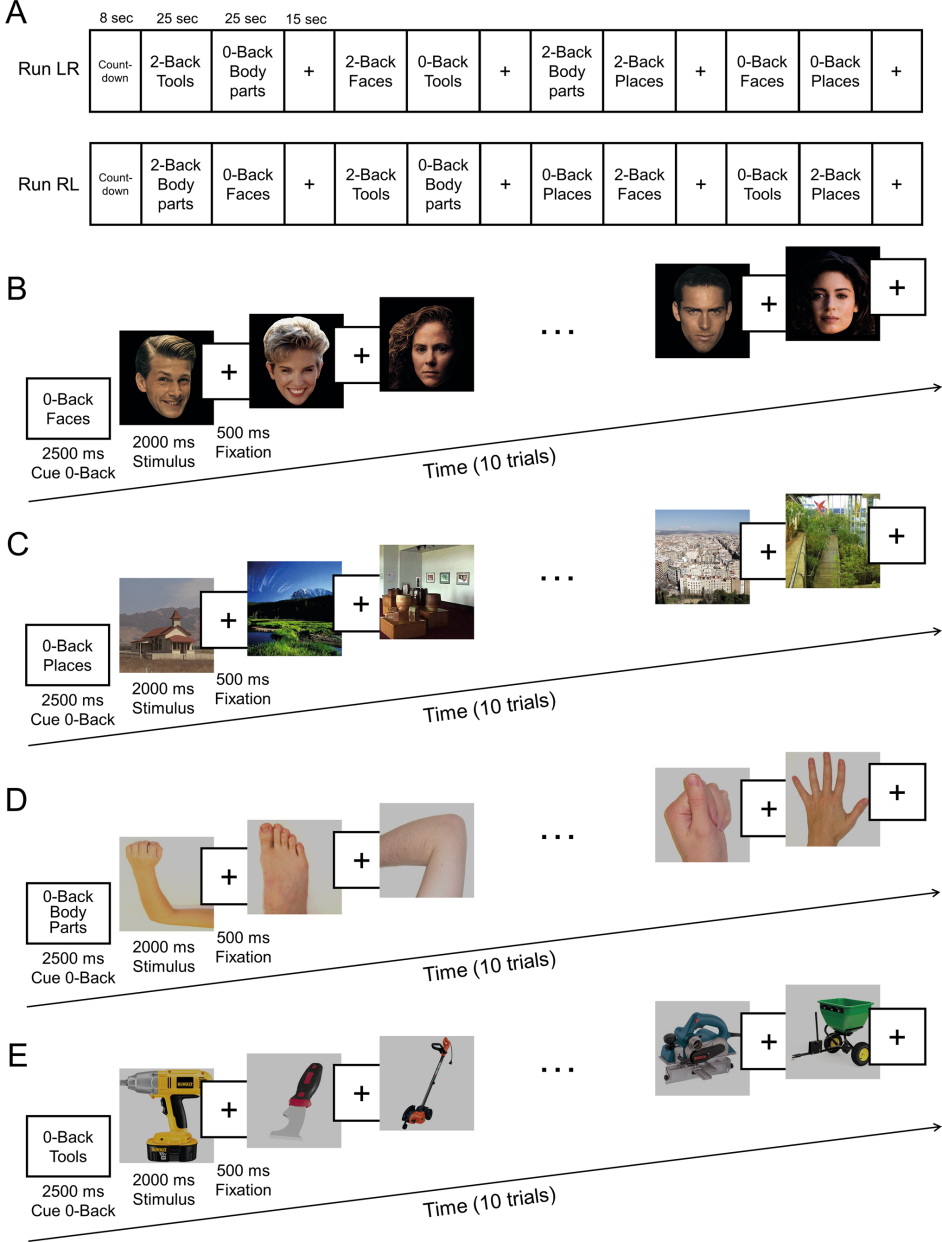
The Human Connectome Project Working Memory task for the 0-back condition (Barch et al. 2013). Four stimulus types were used in a block design, faces, places, tools, and body parts. + indicates a fixation cross presented in the inter-trial interval. Examples of the large set of stimuli used are shown in this figure. In the 0-back condition used for most of the analyses described here, a target cue was presented at the start of each block in the cue period, and the participant had to respond ‘target’ to any presentation of that stimulus in the block. There were 2 runs in which data were acquired, and each run included 8 task blocks, 4 task blocks for 0-back, and 4 task blocks for 2-back. Each stimulus type (faces, scenes etc.) thus had 20 trials as 0-back, and 20 trials as 2-back. **A** The task design in which runs of a task such as the 0-back task were performed. Each run consisted of a 2.5 s cue period followed by 10 trials in which a stimulus was shown for 2 s followed by a 0.5 s fixation period. The 10 stimuli in each run were thus presented over a 25 s period. Each run consisted of either faces, or places or body parts or tools. On 50% of runs, 0-back faces, places and tools were preceded by a 15 s screen showing only a fixation cross. **B**–**E** Examples of the different 0-back runs

The Human Connectome Project (HCP) dataset provides task functional magnetic resonance imaging (fMRI) data for 7 cognitive tasks, one of which is the working memory task (Barch et al. 2013) which provided the data analysed here. In the working memory task, participants were presented with separate task blocks of trials for faces, places, body parts and tools (Barch et al. 2013). Most of the analyses described here were on the 0-back version of the task, illustrated in Fig. 1. The ‘place’ stimuli were views of scenes, and are termed ‘scene stimuli’ here. (Details of the task, and the stimuli used, are available at https://www.humanconnectome.org/hcp-protocols-ya-task-fmri and https://db.humanconnectome.org/app/action/ChooseDownloadResources?project=HCP_Resources&resource=Scripts&filePath=HCP_TFMRI_scripts.zip.) Within each task block, first an instruction image was presented for 2.5 s to indicate the stimulus task type and whether that block was 0-back or 2-back. Then 10 trials were run for a given stimulus type, with each stimulus shown for 2.0 s followed by an interstimulus interval of 0.5 s in which a cross was shown. The 10 stimuli in each block thus lasted for 25 s. In the analyses described here, the activations and functional connectivities were measured as described below during these 25 s periods, which with a TR of 0.72 s provided 35 volumes. There were 2 runs in which data were acquired, and each run included 8 task blocks, 4 task blocks for 0-back, and 4 task blocks for 2-back. Each stimulus type (faces, scenes etc.) thus had 20 trials as 0-back, and 20 trials as 2-back.

### HCP data acquisition

Functional magnetic resonance images (fMRI) were acquired from a large cohort of individuals participating in the working memory task of the HCP (Barch et al. 2013). The data were obtained from the publicly available S1200 release (last updated: April 2018) of the HCP (Van Essen et al. 2013). Participants provided written informed consent, and the scanning protocol was approved by the Institutional Review Board of Washington University in St. Louis, MO, USA (IRB #201204036). In this study, we utilized the task-based fMRI data of the working memory task from all 956 participants who completed both runs of the task with data quality approved by the HCP and who had covariates available.

The whole-brain EPI acquisitions were performed using a 32-channel head coil on a modified 3 T Siemens Skyra scanner. The imaging parameters included a TR of 720 ms, TE of 33.1 ms, flip angle of 52 degrees, bandwidth of 2290 Hz/Px, and in-plane FOV of 208 × 180 mm. Each functional volume comprised 72 slices with a voxel size of 2.0 mm isotropic. A multi-band acceleration factor of 8 was used during image acquisition (Feinberg et al. 2010; Moeller et al. 2010). Two runs of each task were acquired, one with right-to-left phase encoding and the other with left-to-right phase encoding (Barch et al. 2013).

### Calculation of mean BOLD signal level and functional connectivity

The current study employed surface-based timeseries data from the HCP for the working memory task. We parcellated the timeseries data into the 360 cortical regions defined by the surface-based HCP-MMP atlas (Glasser et al. 2016a, b). We extracted the timeseries for each task block which lasted for 27.5 s as described above, using the timing information for each block provided by the HCP (https://www.humanconnectome.org/hcp-protocols-ya-task-fmri).

Within each task block, the BOLD signal showed a consistently high level to the set of stimuli in that task block for the last 20 timepoints in a block (with TR = 0.72 s) (see Fig. S3B), and that period was used for the analysis of the responses to the stimuli. The calculation of the average BOLD signal level for each cortical region for each stimulus type for 0-back was averaged for each subject across the two runs. The same procedure was used for the 2-back.

Additionally, the FC matrices for each participant were constructed by assessing the Pearson correlation between the last 20 timepoints of the timeseries for the 180 cortical regions in each hemisphere (again using the mean between the two runs available for each stimulus type for both 0-back and 2-back).

### Statistical analysis

We have just described how the data for individual subjects were extracted. For the population-based statistical analysis, the aim was to examine the selectivity of the activations for each of the four stimulus types, faces, scenes, body parts, and tools. To implement this, paired t-tests were performed to examine the differences in the mean BOLD signal level between each stimulus type condition and the mean of the BOLD signal for the four stimulus type conditions for each of the 180 cortical regions in a hemisphere, using Bonferroni correction for multiple comparisons. The covariates of no interest including sex, age, drinker status, smoking status, education qualification, and head motion, were regressed out of this analysis. Separate activation maps were calculated for the left and right hemispheres.

For the Functional Connectivities, paired t-tests were conducted to identify FCs that were selective for each stimulus type condition, by comparing FCs between each condition and the mean of the four conditions. FDR correction was applied to account for multiple comparisons, with the above covariates of no interest regressed out. FDR correction was used, given that each functional connectivity matrix was 180 × 180. The effect size, measured with Cohen's d, was calculated as the number of standard deviations between the means of the two conditions. The results are presented separately for the 180 × 180 FC matrices for the left and right hemispheres.

The selective activations and FCs for each stimulus type (faces, scenes, body parts and tools) were calculated here as the signal (or FC) for each stimulus type—the mean for the four stimulus types.

We felt it useful to also provide the response for each cortical region to the mean of the four visual stimulus types. To do this, we calculated across participants a t-value contrast for the response period noted above in each timeseries (the last 20 timepoints), and compared this to the lower signal in the first 15 timepoints of each timeseries. The results of this analysis are shown in Fig. S3A. The mean activations across faces, scenes, body parts and tools were high in the regions shown in Fig. S3A, and included visual and related cortical regions (Rolls et al. 2023b) V1–V4; ventromedial visual cortical stream regions DVT and ProS [where the retrosplenial scene area is located (Sulpizio et al. 2020)] VMV1-3, VVC, and medial parahippocampal PHA1-3 [where the parahippocampal scene area is located (Sulpizio et al. 2020)]; the ventrolateral cortical stream regions PIT, FFC, PH to TE1p and TE2p, with their onward connectivity to lateral parahippocampal cortex TF and perirhinal cortex; some superior temporal cortex visual stream activation (STSdp); some semantic regions (TPOJ1-3) (Rolls et al. 2022a); some dorsal visual stream (MT, MST, FST) and connected intraparietal regions (AIP, LIPv, LIPd, IP2, IP1); some inferior prefrontal cortex regions (IFJp, IFJa, IFSp, IFSa); and eye field regions (FEF, PEF, SCEF). The selective activations reported in this paper were increases to each of faces, scenes, body parts, or tools above these mean activations to all four stimulus types.

For completeness, Fig. S3D shows the cortical regions with significant selective differences in the activations for faces, places, tools and body parts when the baseline was the mean activation for the other three stimuli, a type of baseline that has been used previously (Grill-Spector et al. 1998; Stigliani et al. 2015; Natu et al. 2019; Nordt et al. 2021).

## Results

### Activations by faces, scenes, tools and body parts

**Fig. 2:**
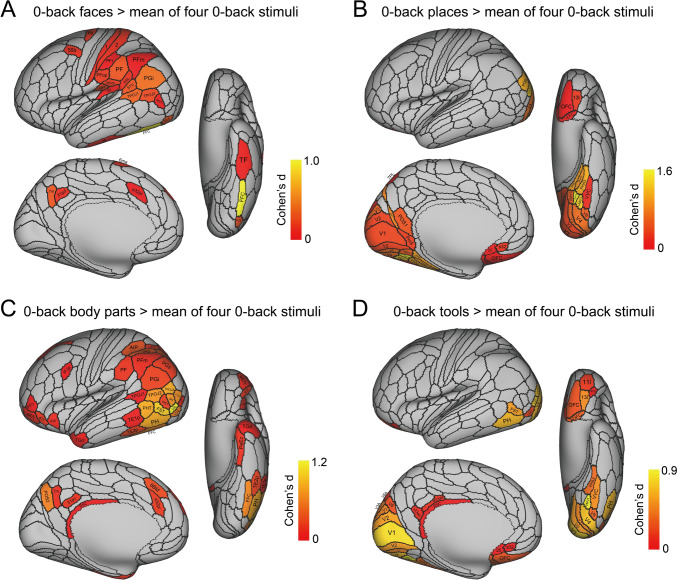
Brain regions in the left hemisphere with significant differences in the average BOLD signal for the faces, scenes, body parts, and tools compared to the mean of these conditions in the 0-back working memory task, after Bonferroni correction (α = 0.05). Panels **A**, **C** show the top 30% of brain regions with significant differences for the 0-back faces and 0-back body parts conditions contrasted with the mean of the four conditions, respectively. Panels **B**, **D** display all the brain regions with significant differences for the 0-back scenes and 0-back tools conditions compared to the mean of these four conditions, respectively. The selection of the top 30% of cortical regions in **A** and **C** allows the main differences between the four stimulus type, faces, places, body parts, and tools to be easily visualised, but for completeness Fig. S3C shows the same figure as this but without any selection of the top 30%. The corresponding figure for the right hemisphere is in Fig. S2

**Fig. 3:**
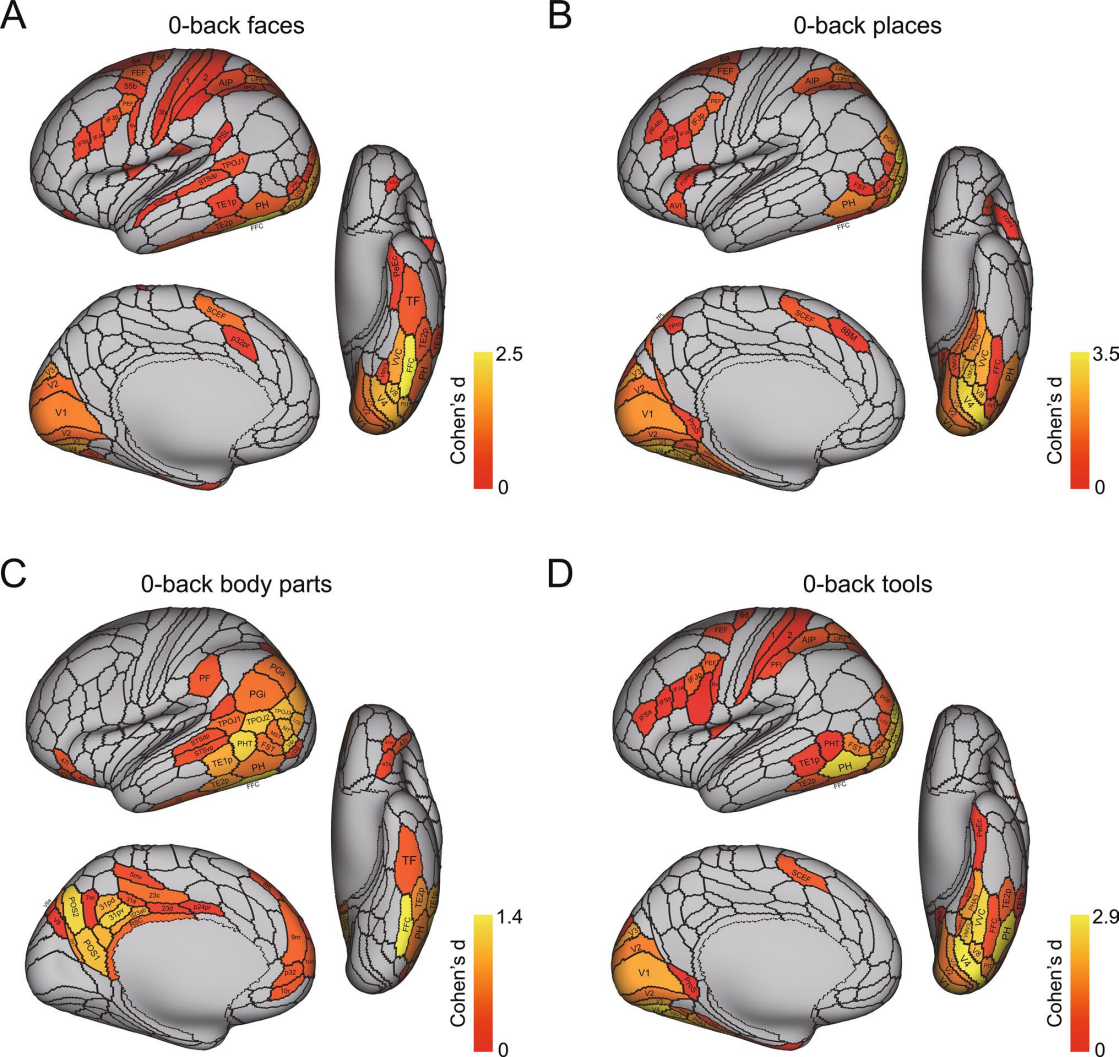
The cortical regions exhibiting significant differences in the average BOLD signal between the baseline prestimulus period preceding the 0-back blocks (shown in Fig. 1) before the BOLD signal had responded to the stimuli, and the last 20 timepoints within the 0-back blocks (when the BOLD signal response to the visual stimuli was occurring) for each of the four stimulus types (faces, places, body parts, and tools) after Bonferroni correction (α = 0.05) across 956 participants. The effect size as indicated by Cohen’s d is indicated. The activations are shown in red to yellow. The top 50 cortical regions with significant increases in the BOLD signal are shown out of the 180 cortical regions in the left hemisphere. The baseline prestimulus period was for the last 5 s of the 15 s fixation time and the initial 15 timepoints with a TR of 0.72 s starting when the cue was shown in a run (see Fig. 1)

The activations to each of faces, scenes, tools, and body parts are shown for each as the difference from the mean activation to all four stimulus types in Fig. 2 on the HCP-MMP left hemisphere. The results are shown for the right hemisphere in Fig. S2. A list of the abbreviations for the cortical regions is provided in Table S1. The results are shown for the 0-back condition to minimize the memory load, so as to reveal differences in the activations for these different types of stationary visual stimuli. The results reported below shown in Fig. 2 are supported by an analysis in which the baseline for each stimulus type was the mean of the activations to the other three stimuli (Fig. S3D), a type of baseline that has been used previously (Grill-Spector et al. 1998; Stigliani et al. 2015; Natu et al. 2019; Nordt et al. 2021). The results are also supported by an analysis in which the activation to each stimulus type (e.g. scenes) was compared to the prestimulus baseline, which enables activation to e.g. scenes to be shown with no effect related to other stimuli (Fig. 3). The advantage of what is provided in Figs. 2 and S2 is that these show activations selective to each category (e.g. scenes) compared to the same mean baseline computed across all of the stimuli shown in the investigation.

#### Faces

Figure 2a (see also Table S2, and Fig. S2 panel A) shows the highest selective activation for the sight of faces in region FFC, with activations too in adjacent and connected TF which is the lateral parahippocampal cortex with connectivity to the hippocampal memory system (Huang et al. 2021; Ma et al. 2022; Rolls et al. 2022b). Interestingly, visual inferior parietal regions PGi, PGs and PFm which have connectivity with anterior temporal lobe visual and semantic regions (Rolls et al. 2023e) were also activated, as consistently were language-related cortical regions TPOJ1, TPOJ2, PSL (the PeriSylvian Language region), STV (the Superior Temporal Visual), and 55b (Rolls et al. 2022a). Also interestingly, inferior parietal high order somatosensory regions PF, PFt, PFop, PFcm and earlier somatosensory regions in the insula (RI), operculum (OP1) (Rolls et al. 2023d) and somatosensory cortical regions 1 and 2 were selectively activated by the sight of faces. Comparable activations were found for the right hemisphere as illustrated in Fig. S2 panel A (see also Table S4), with the FFC region strongly activated by faces, and also the superior temporal sulcus (STS) cortical regions in which we discovered that neurons respond to faces (Baylis et al. 1987; Hasselmo et al. 1989a, b; Rolls 2024a).

#### Scenes/places

Figure 2b (see also Table S2, and Fig. S2 panel B) shows selective activations to viewed scenes (termed ‘places’) in the ventromedial visual regions VMV1, VMV2, VMV3, VVC, and their forward connected medial parahippocampal cortical regions PHA1, PHA2, PHA3 [where the parahippocampal place or scene area is located (Sulpizio et al. 2020)] which in turn have connectivity into the hippocampal memory and navigation system (Rolls et al. 2023b; Rolls et al. 2023a, b, c, d, e, f, g, h). Activations in DVT and the ProStriate Cortex ProS [where the retrosplenial scene area is located (Sulpizio et al. 2020)], and which projects to the VMV regions (Rolls et al. 2023b; Rolls et al. 2023a, b, c, d, e, f, g, h; Rolls et al. 2024a, b), are also evident, as is activation in POS1 which has high effective connectivity with ProS (Rolls 2024a). Strong selective activation was also found in inferior parietal region PGp, which has connectivity to the hippocampal memory and navigation system and may be involved in self-motion update of scene representations (Rolls 2023a; Rolls et al. 2023e). Activation in earlier cortical regions (V1, V2, V3, V4) is also evident with this contrast (see also Fig. 3 panel B). Interestingly, these scene visual stimuli also selectively activated the medial orbitofrontal cortex (OFC, 13 l and pOFC) and related anterior cingulate regions s32 and 25 which represent reward value (Rolls 2023b; Rolls et al. 2023c) when the baseline was the activation to all 4 stimuli. Comparable activations were found for the right hemisphere as illustrated in Fig. S2 panel B (see also Table S4).

#### Body parts

Figure 2c (see also Table S2, and Fig. S2 panel C) shows selective activations to the sight of body parts in visual inferior parietal regions PGi, PGs and PFm, and in visual posterior inferior temporal visual cortical regions FFC, PH, PHt, TE1p and TE2p (Rolls et al. 2023e). Visual motion regions such as MT, MST and FST were also activated by the sight of (stationary) body parts. Parietal regions AIP, IP2, and LIPd involved in eye movement control and visually guided actions in space (Rolls et al. 2023b) were also selectively activated by the sight of body parts. Inferior parietal somatosensory region PF at the top of the somatosensory hierarchy (Rolls et al. 2023e) was also activated. Language-related cortical regions TPOJ1, TPOJ2, TPOJ3 and TGd (Rolls et al. 2022a) were also activated. The perirhinal cortex, a route for object information to reach the hippocampal memory system (Rolls et al. 2023b), and also parts of the posterior cingulate cortical division (31pv, 7 m) were also activated. Lateral orbitofrontal cortex regions a47r, p47r and 47 l were also activated by the sight of body parts, which may be related to these stimuli being somewhat unpleasant as some look like dismembered limbs (see Fig. 1), as the lateral orbitofrontal cortex is activated by unpleasant stimuli (Grabenhorst and Rolls 2011; Rolls 2019a, 2023b). Comparable activations were found for the right hemisphere as illustrated in Fig. S2 panel C (see also Table S4), though were less evident in the inferior parietal cortex when selecting only the top 30% of regions with significant activations.

#### Tools

Figure 2d (see also Table S2, and Fig. S2 panel C) shows selective activations to the sight of tools in the lateral parts of the ventromedial visual regions VMV3, VVC, and PHA3, and in visual motion regions V6, V6a, FST and PH. There is also activation evident in earlier cortical visual regions V1, V2, V3 and V4, and also some posterior cingulate division regions including RSC, v23ab, and d23ab. Tools also activated the medial orbitofrontal cortex and related reward regions, perhaps reflecting that tools are associated with goal/reward-related actions. Comparable activations were found for the right hemisphere as illustrated in Fig. S2 panel D (see also Table S4), with again the lateral parts of the ventromedial visual cortical stream (Rolls 2024a), including VMV3, VVC and PHA3 strongly activated by the sight of tools.

#### Activations for faces, scenes, body parts and tools shown against a pre-stimulus baseline

Figure 2 shows the selective activations for each stimulus type, faces, places (scenes), body parts, and tools, using as a baseline the mean of the activations across all four stimulus types. To complement this analysis, and in order to show the cortical regions activated by each stimulus type independently of any other stimulus type, Fig. 3 shows the cortical regions with significant responses separately for faces, places, tools and body parts, where the responses are measured as a significant difference in the BOLD signal between the response period (the last 20 timepoints in the timeseries), compared to the lower signal in the prestimulus baseline during the last 5 s of the 15 s fixation period and the first 15 timepoints of each timeseries for each run (see Figs. 1 and S3B). The contrasts in this analysis were thus for faces—the prestimulus baseline, places—the prestimulus baseline, etc., and thus the activations shown are those produced only by faces, or by places, or by tools, or by body parts. There was in this analysis little influence of other stimuli on the activations to faces, places, etc. Given the TR of 0.72 s and the haemodynamic response function, use of the last 5 s of the fixation period and the first 15 time points starting when the cue for a start of a run was shown was appropriate and no activation was evident in this time period, as shown in the timecourse in Fig. S3B. Given that there were typically 39 timepoints in each run, the last 20 did show clear activations to the stimuli, as shown in Fig. S3B. Similar results were found if bins 6–16 in the timecourse in Fig. S3B were used as the prestimulus baseline.

The results shown in Fig. 3 help to confirm the findings shown in Fig. 2. For example, Fig. 3a shows that faces activate strongly FFC; moderately V4, V8, VVC, and TF; and to some extent TF, PeEc, TE2p, STSda, STSdp, TPOJ1-3, and the somatosensory cortex (3b, 1, 2).

Figure 3b shows that places (scenes) activate strongly V4, VMV1, VMV2; moderately PHA1, PHA2, PHA3, VVC, PH and PGp; and to some extent regions where the retrosplenial scene area is located ProStriate (ProS), and the dorsal visual transitional area DVT (Sulpizio et al. 2020).

Figure 3c shows that body parts strongly activate FFC, moderately activate PH, TE2p, TE1p, MT, MST and FST; and to some extent regions STSdp and STSvp, PF, PGi and PGs, and TOPJ1-3.

Figure 3d shows that tools strongly activate VVC, VMV2, V8, V4 and PH; moderately activate PHA3 and FST; and activate to some extent somatosensory 1, 2 and PFt.

Figure 3 helps to emphasise a gradient of efficacy of stimuli from medial to lateral in the ventral temporal lobe, with scenes most medial, then moving laterally tools, then faces, and then most lateral body parts.

### Functional connectivities for faces, scenes, tools and body parts

**Fig. 4:**
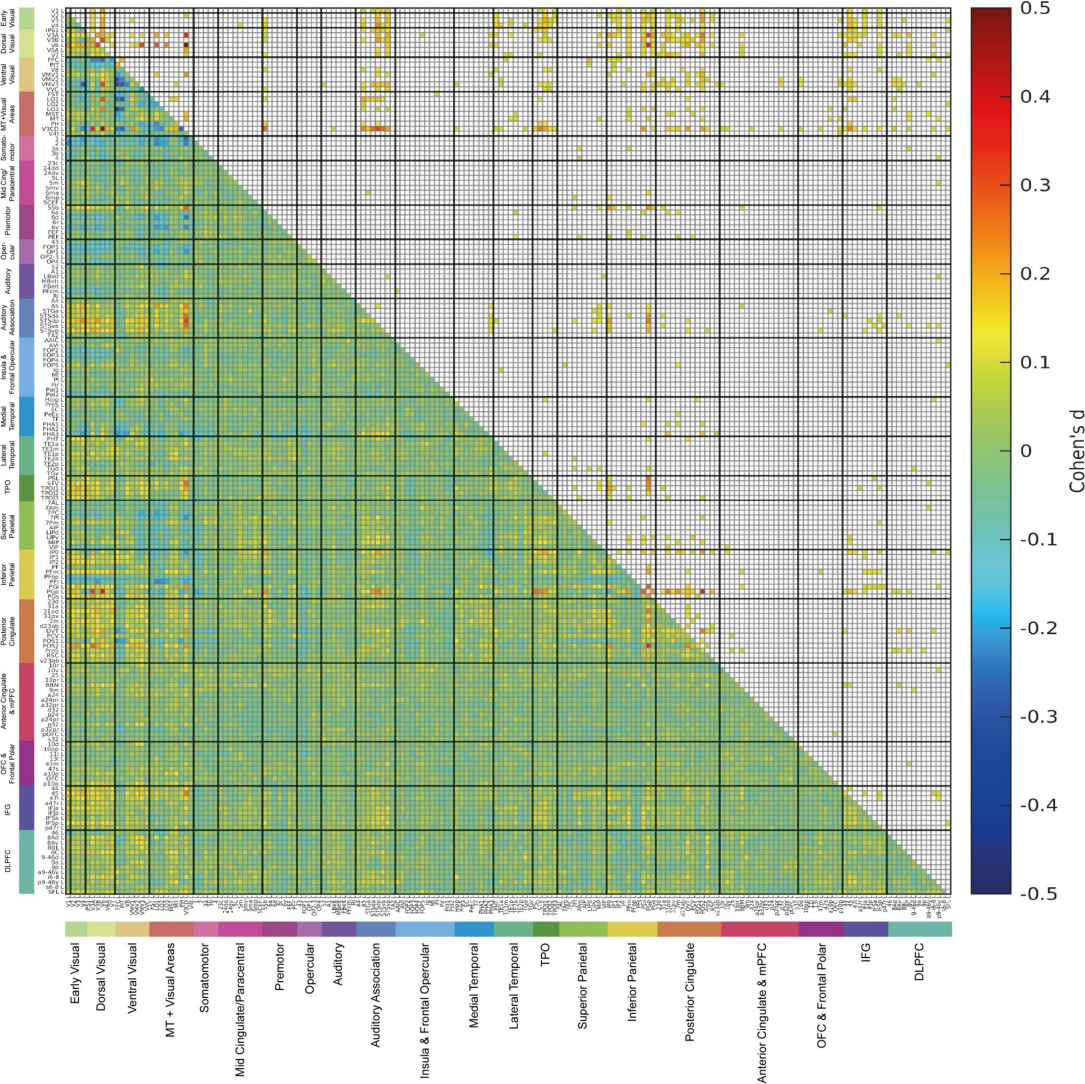
The lower left triangle shows the matrix of functional connectivity differences between 0-back faces and the mean of all 0-back conditions with the Cohen’s d values showing the effect size of the differences. The matrix is for the functional connectivities in the left hemisphere, as listed in Table S1, with V1, V2, V3 … at the top of the y axis and the left of the x axis. The upper triangle matrix shows the Cohen’s d values of positive significant links after FDR correction (α = 0.05). These results were from 956 participants in the HCP dataset. All the values shown in the matrix were limited to the range from − 0.5 to 0.5. The covariates regressed out in this analysis were sex, age, drinker status, smoking status, education qualification and head motion

**Fig. 5:**
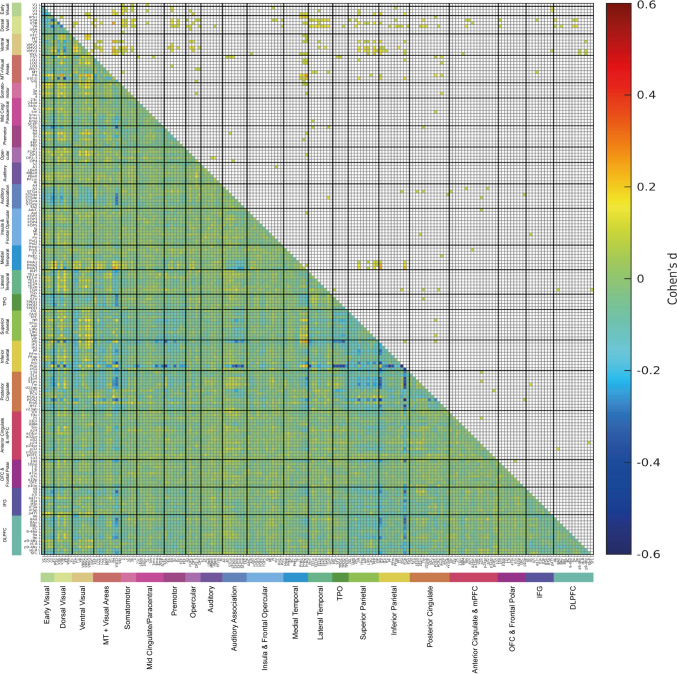
The lower left triangle shows the matrix of functional connectivity differences between 0-back scenes and the mean of all 0-back conditions with the Cohen’s d values showing the effect size of the differences. The matrix is for the functional connectivities in the left hemisphere, as listed in Table S1, with V1, V2, V3 … at the top of the y axis and the left of the x axis. The upper triangle matrix shows the Cohen’s d values of significant positive links after FDR correction (α = 0.05). These results were from 956 participants in the HCP dataset. All the values shown in the matrix were limited to the range from − 0.6 to 0.6. The covariates regressed out in this analysis were sex, age, drinker status, smoking status, education qualification and head motion

**Fig. 6:**
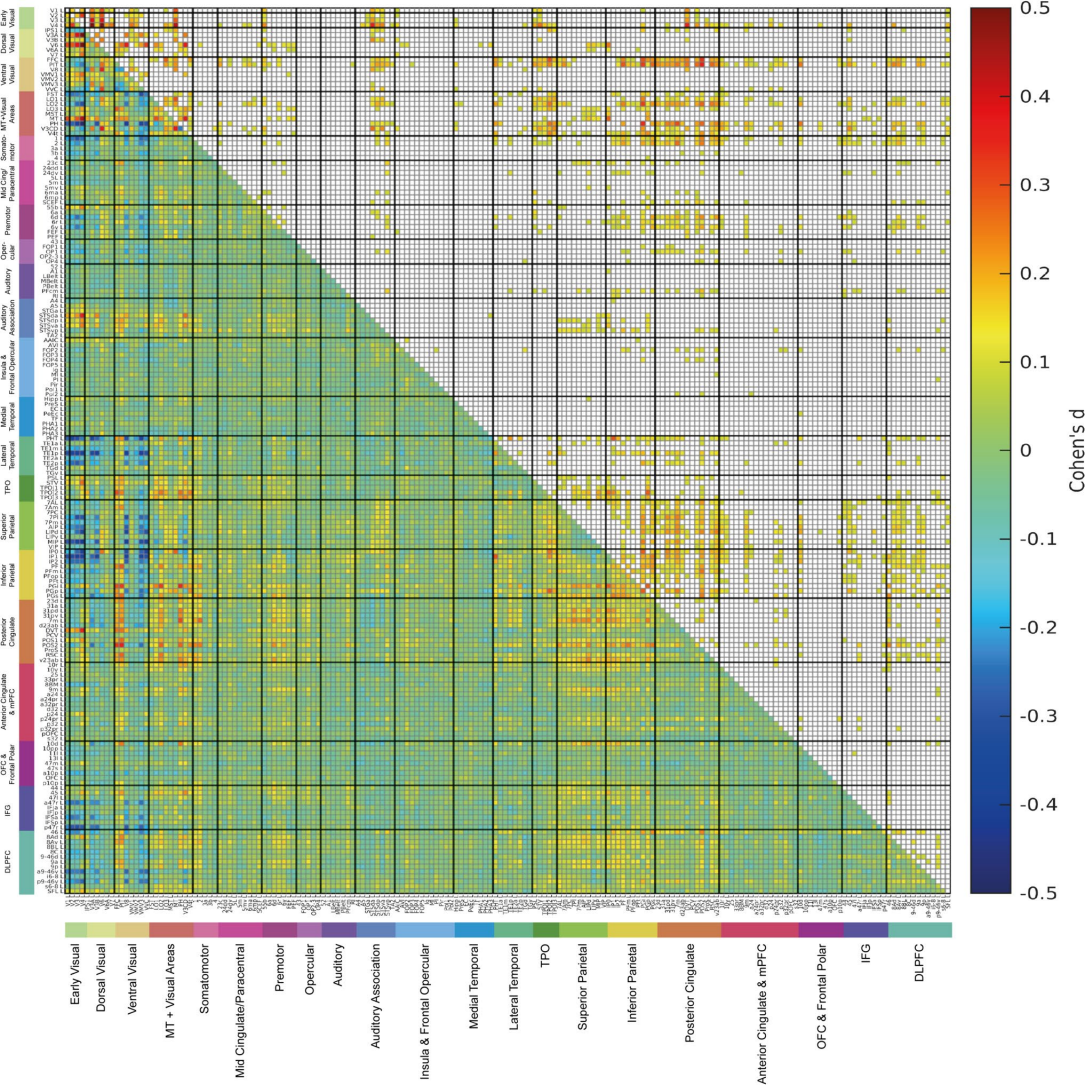
The lower left triangle shows the matrix of functional connectivity differences between 0-back body parts and the mean of all 0-back conditions with the Cohen’s d values showing the effect size of the differences. The matrix is for the functional connectivities in the left hemisphere, as listed in Table S1, with V1, V2, V3 … at the top of the y axis and the left of the x axis. The upper triangle matrix shows the Cohen’s d values of significant positive links after FDR correction (α = 0.05). These results were from 956 participants in the HCP dataset. All the values shown in the matrix were limited to the range from − 0.5 to 0.5. The covariates regressed out in this analysis were sex, age, drinker status, smoking status, education qualification and head motion

**Fig. 7:**
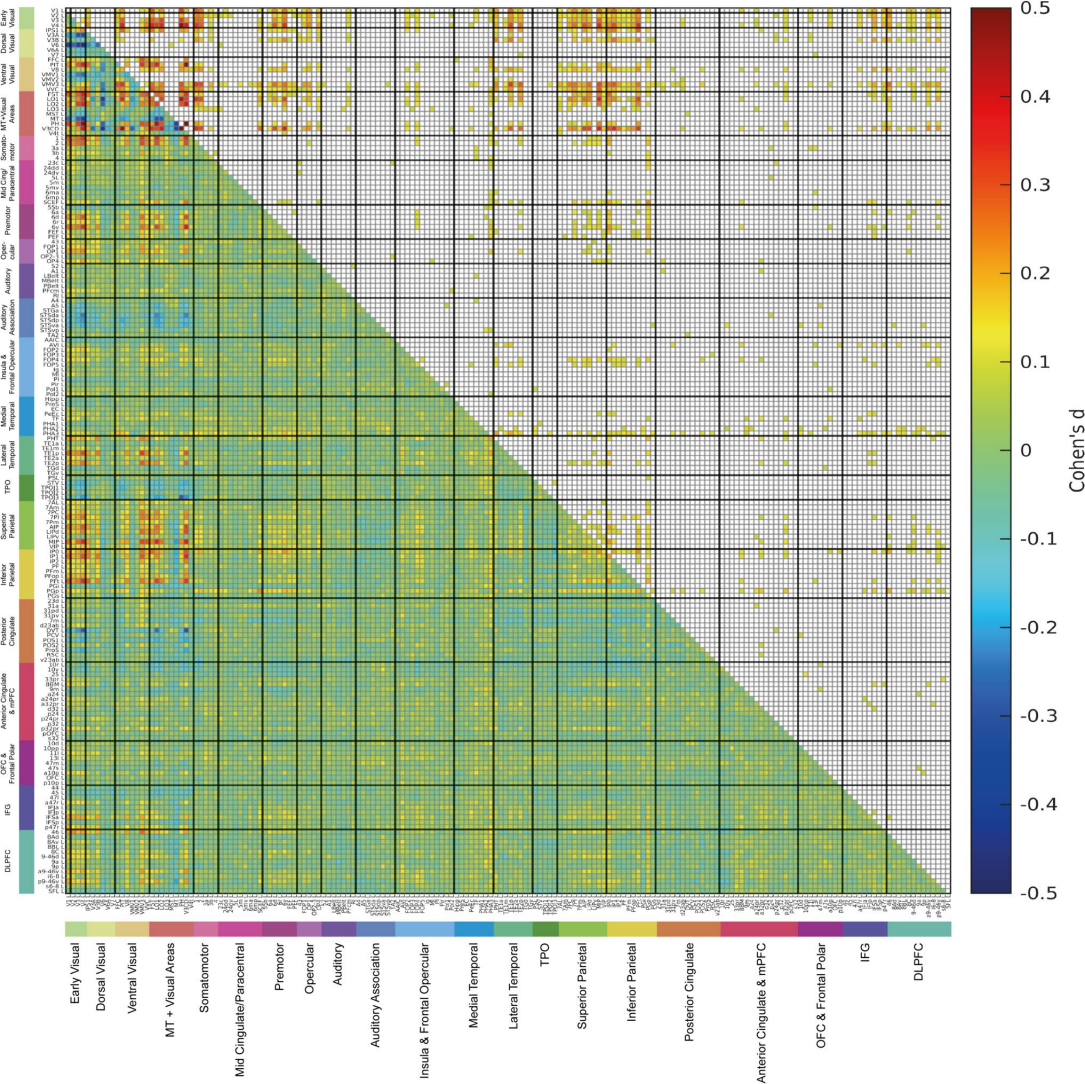
The lower left triangle shows the matrix of functional connectivity differences between 0-back tools and the mean of all 0-back conditions with the Cohen’s d values showing the effect size of the differences. The matrix is for the functional connectivities in the left hemisphere, as listed in Table S1, with V1, V2, V3 … at the top of the y axis and the left of the x axis. The upper triangle matrix shows the Cohen’s d values of significant positive links after FDR correction (α = 0.05). These results were from 956 participants in the HCP dataset. All the values shown in the matrix were limited to the range from − 0.5 to 0.5. The covariates regressed out in this analysis were sex, age, drinker status, smoking status, education qualification and head motion

The functional connectivity differences for each of faces, scenes, tools, and body parts are shown for each as the difference from the mean functional connectivity across all four stimulus types in Figs. 4, 5, 6 and 7 on the HCP-MMP left hemisphere, with the corresponding results for the right hemisphere in Figs. S4–S7. As the results are shown for each stimulus type relative to the mean across all stimuli, the higher functional connectivities in each of these figures show what is selective for each of the 4 stimulus types. The results are shown for the 0-back condition to minimize the memory load, so as to reveal differences in the functional connectivities for the different types of stimuli.

A key point to emphasise is that the functional connectivities to these four stimulus types are different to each other, and different from the functional connectivities in the resting state (Rolls et al. 2023b, 2023e, 2023b), providing evidence that functional connectivities change depending on the task, and providing evidence about pathways especially involved in particular tasks. Although resting state functional connectivity is a useful measure of the basic framework of brain connectivity, the connectivity is not in the short term fixed like anatomical connectivity, but provides evidence about cortical pathways involved in particular functions. An indication of how the functional connectivities can change when visual stimuli are presented is provided in Figs. S8 and S9, which show which functional connectivities increase when visual stimuli are shown compared to the pre-stimulus period.

#### Faces

The functional connectivities selective for faces are those significantly greater than the mean across all visual stimuli shown in the upper right triangle of Fig. 4, with the positive Cohen’s d values showing the effect size. Higher functional connectivities for faces are found for STS and related regions (such as STGa, STSda, STSdp, STSva, STSvp) with some early visual cortical regions, including V2, V3, V4; with some ventral stream regions including V8, and VMV3 just medial to the FFC; some dorsal stream regions including IPSI, V3A, V3B, and V6; some MT + complex regions including LO1 and V3CD; intraparietal LIPv, MIP and IP0; and inferior parietal PGp. These connectivities are very interesting, for many neurons in the cortex in the macaque STS respond preferentially to moving faces that make or break social contact (Hasselmo et al. 1989a, b), and inputs from both ventral stream face/object and dorsal visual stream movement-related regions are likely to implement this (Baylis et al. 1987; Hasselmo et al. 1989a, b; Hasselmo et al. 1989a, b). Many of the visual regions just noted also have significantly high functional connectivities when viewing faces with inferior parietal regions (such as PGi, PGs and especially PGp) (Rolls et al. 2023e); with posterior cingulate division regions (including PCV—the precuneus visual region, POS1, POS2, 23d, 31a, 31pd) (Rolls et al. 2023b); and with region 45, part of Broca’s area. Connectivity in the right hemisphere was similar, with in addition higher connectivity of some of these visual cortical areas with temporo-parietal junction semantic regions PSL, STV, TPOJ1 and TPOJ2; and with some somatomotor regions including 3b and 4; and of V3CD with A1 and belt auditory regions (Fig. S4).

#### Scenes/places

The functional connectivities selective for places (i.e. views of spatial scenes) shown in Fig. 5, and especially Fig. S5 for the right hemisphere, were higher for ventromedial visual regions VMV2 and VMV3 with ventral visual stream regions V3, V4, FFC, PIT and V8. The parahippocampal scene area (or parahippocampal place area PPA) is at the junction of the VMV and medial parahippocampal PHA regions (Sulpizio et al. 2020), and this connectivity finding supports the theory that in humans and other primates scene representations are built using ventral stream feature combination mechanisms (Rolls 2023a; Rolls and Treves 2024). Consistent with this, the parahippocampal regions PHA1, PHA2 and PHA3 regions have high functional connectivity with similar ventral stream regions (V3, V4, FFC, PIT, V8) during the visual presentation of scenes (places) (Fig. S5). Interestingly, higher functional connectivity during scene viewing was also found for connectivities of VMV2, VMV3 and VVC with PHA1-3 regions and with some visual motion-related regions: MT + regions such as FST, LO1, LO2, PH, V3CD and V4t; regions in the superior parietal cortex (7Pl, 7Pm) and intraparietal cortex (LIPd, MIP, IP0); and inferior parietal cortex (PGp). This supports the hypothesis that these visual motion-related dorsal stream regions that reach the parietal cortex provide a self-motion update for where the observer is viewing in a visual scene (Rolls 2023a).

#### Body parts

The functional connectivities selective for body parts shown in Fig. 6, and Fig. S6 for the right hemisphere, have significant selectively high functional connectivity for early visual cortical regions V1, V2, V3 and V4, and/or ventral stream regions FFC, PIT and V8 with STSda, STSdp and STSva [where body parts as well as faces are represented in macaques Perrett et al. 1985; Rolls and Tovee 1995)]; with temporo-parietal junction semantic regions (PSL, STV, TPOJ1-3) (Rolls et al. 2022a); with inferior parietal cortex regions (the regions with primarily somatosensory inputs PF, PFop, PFt, and the regions with primarily visual inputs PGi, PGp, PGs and PFm) (Rolls et al. 2023e); and most regions in the posterior cingulate division (Rolls et al. 2023b). Visual movement-related regions including some MT + regions (especially LO2, PH and V4t, but also LO1 and MT) and superior parietal area 7 and intraparietal regions also have significantly selectively high functional connectivity with similar cortical regions to those described, and interestingly with lateral temporal regions especially PHT, TE1p and TE2p (the latter two being the last primarily visual cortical regions in the ventrolateral visual cortical stream (Rolls et al. 2023a, b, c, d, e, f, g, h). Thus some STS regions, temporo-parietal junction regions, and inferior parietal cortex regions have selectively high functional connectivity with both ventral visual stream and dorsal visual stream (MT + , intraparietal, area 7) cortical regions during viewing of body parts, and consistent with this, these brain regions are likely to be involved in representing body parts often when they are moving, and hence are likely to be important in perceiving the meaning of movements of body parts (STS and temporo-parietal junction regions) (Perrett et al. 1985; Jellema and Perrett 2003b, a; Rolls et al. 2023b), and in performing actions in space with body parts (inferior parietal regions) (Rolls et al. 2023e). Interestingly, there is also selectively high functional connectivity of somatosensory cortex regions 1 and 2 not only with some MT + and intraparietal cortex visual motion regions, but also with some of the inferior parietal (PF, PGi, PGs) and posterior cingulate division regions during viewing of body parts, reflecting multimodal processing that includes somatosensory in these visual motion, inferior parietal and posterior cingulate division regions when stationary visual stimuli of body parts are being viewed (Figs. 6 and S6).

#### Tools

The functional connectivities selective for tools shown in Figs. 7 and S7 reveal selective high connectivity of early cortical visual areas V1, V2, V3, V4, and ventral stream V8, and lateral parts of the ventromedial visual stream VMV3 and VVC with visual inferior temporal cortex PHT, TE1p and TE2p; with intraparietal and superior parietal area 7 regions; with inferior parietal regions PF, PFop and PFt that have primarily somatosensory inputs as well as with PGi, PGs and PFm that have visual inputs (Rolls et al. 2023e). The pathway for tools to the inferior temporal visual cortex is likely to be involved in object recognition for tools; to provide visual form information to the intraparietal and superior parietal regions involved in visually guided actions in space; and to the inferior parietal visual and somatosensory regions to provide multimodal somatosensory and visual object recognition and use of tools (Rolls et al. 2023b, 2023e). However, visual motion inputs from V3B, and from MT + regions FST, LO1, LO2, PH and V3CD, also have selective connectivity with these same intraparietal, superior parietal area 7 and inferior parietal regions, which are likely to be important for perceiving tools which often have characteristic motions, and for performing visually guided actions with tools. It is of interest that in contrast to faces, viewing tools did not increase functional connectivity in STS regions, but did in inferior parietal cortex regions.

### Cortical regions activated by increasing the memory load for faces, scenes, body parts, and tools from 0-back to 2-back

**Fig. 8:**
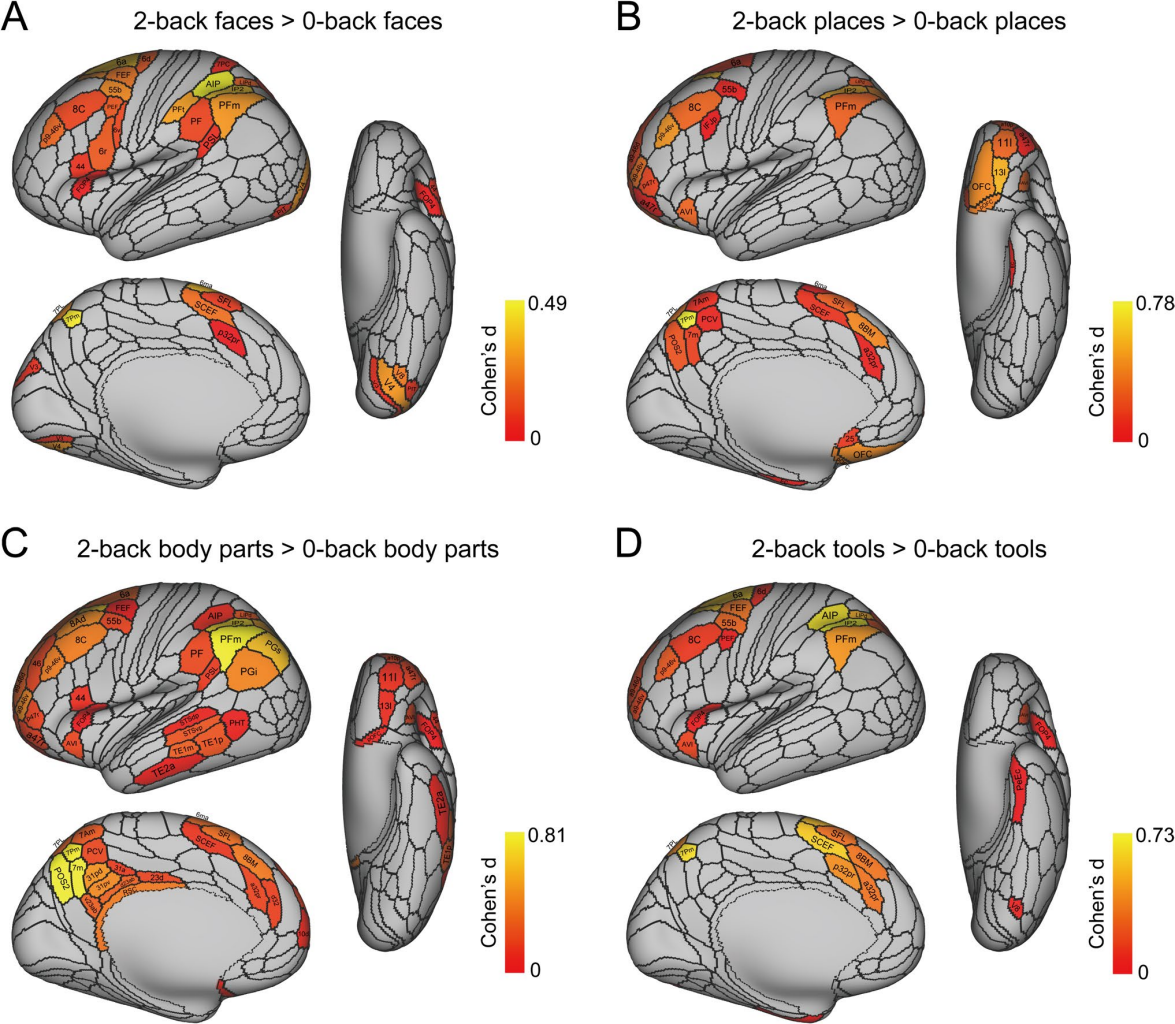
Brain regions showing significant differences in the average BOLD signal between the four 2-back working memory conditions and their corresponding 0-back conditions, after Bonferroni correction (α = 0.05). Panels **A**–**C** display brain regions with significant differences for the 2-back faces, 2-back scenes and 2-back body parts conditions, contrasted with their corresponding 0-back conditions, respectively. Panel **D** shows brain regions with significant differences for the 2-back tools condition compared to the 0-back tools condition

To reveal whether increasing the memory load for faces, scenes, body parts and tools increases the activation in the cortical regions that represent these visual stimuli, or in other cortical regions, we compare in Fig. 8 the activations for the contrast 2-back versus 0-back for each of these four types of stimuli (see also Table S6). An overview is that activations increase mainly in cortical regions other than those in which these stimuli are represented. The regions that are recruited include especially prefrontal cortex regions implicated in working memory, but also for all stimulus types more activation in some inferior parietal cortex regions (especially PFm), and in some cases posterior cingulate division and other cortical regions.

For faces, Fig. 8a shows that the additional cortical regions recruited for 2-back minus 0-back include prefrontal cortical regions 8C and p9-46v; inferior parietal regions PFm, PF, PFt and some related parietal regions including AIP and 7Pm; V4 and V8; and some premotor and frontal eye field regions that are implicated in working memory (Goldman-Rakic 1996).

For scenes, Fig. 8b shows that the additional cortical regions recruited for 2-back minus 0-back include prefrontal cortical regions 8C, p9-46v, a9-46v and IFJp; inferior parietal PFm; and posterior cingulate division regions.

For body parts, Fig. 8c shows that the additional cortical regions recruited for 2-back minus 0-back include prefrontal cortical regions 8C, p9-46v, a9-46v and also the more dorsal prefrontal regions 46 and a9-46d; inferior parietal PFm, PGs, PGi and PF; intraparietal AIP, LIPd and IP2; temporal lobe regions in the superior temporal sulcus (STSdp, STSvp), in the visual posterior inferior temporal cortex (PHt and TE1p), and in the anterior temporal lobe semantic regions (TE1m, TE2a); and in the posterior cingulate division regions.

For tools, Fig. 8d shows that the additional cortical regions recruited for 2-back minus 0-back include prefrontal cortical regions 8C, p9-46v, a9-46v and also the more dorsal prefrontal regions 46 and a9-46d; inferior parietal PFm, PGs, PGi and PF; intraparietal AIP, LIPd and IP2; temporal lobe regions in the superior temporal sulcus (STSdp, STSvp), in the visual posterior inferior temporal cortex (PHt and TE1p), and in the anterior temporal lobe semantic regions (TE1m, TE2a); and in the posterior cingulate division regions.

### Activations in hippocampal memory-related regions

Although the 0-back and 2-back memory tasks are primarily short-term memory tasks that engage prefrontal cortex regions as described in the preceding section, these visual stimuli did produce some activations of hippocampal system regions, and these are reported in Tables S3 and S5. In the Left hemisphere (Table S3), the hippocampus, entorhinal cortex, and perirhinal cortex and lateral parahippocampal cortex TF were activated more by visual stimuli of body parts than by faces, scenes and tools. Also in the Left hemisphere, the scene regions (VMV1-3, VVC, PHA1-3) were more strongly activated by scenes than the other 3 types of visual stimuli. In the Right hemisphere (Table S5), the hippocampus, entorhinal cortex, and perirhinal cortex were activated more by visual stimuli of faces than by body parts, scenes and tools. Also in the Right hemisphere, the scene regions (VMV1-3, VVC, PHA1-3) were more strongly activated by scenes than the other 3 types of visual stimuli.

## Discussion

The selective activations and functional connectivity increases to visual stimuli of faces, scenes, body parts and tools revealed here help by adding function to the analyses of cortical connectivity using diffusion tractography, and resting state functional and effective connectivity (Rolls et al. 2023b, 2023e, 2023b), in the same Human Connectome Project Cortical parcellation atlas. This is a fundamental step forward because it helps to add functions to the cortical connectivity maps already described, to build a better understanding of cortical function. It was shown here that faces (compared to the mean of all four stimulus types) activated and increased functional connectivities not only in ventrolateral visual stream regions leading to the FFC and lateral parahippocampal gyrus region TF; but also to cortical regions in the STS that receive both ventral stream and dorsal stream inputs to enable representations to be built of moving heads and faces that are socially relevant; and to visual inferior parietal regions such as PGi, PFm that appear to be part of a semantic system extending from these inferior parietal regions through temporo-parietal regions to anterior inferior temporal lobe and temporal pole regions (Rolls et al. 2022a). Scene stimuli [sometimes termed ‘place’ (Epstein and Kanwisher 1998)] selectively activate a ventromedial cortical stream involving ventromedial (VMV) and medial parahippocampal (PHA) regions. Body parts activate a lateral inferior temporal cortical stream leading to posterior inferior temporal visual cortex (FFC, PH, TE2p) and via the perirhinal cortex to the hippocampal memory system, but also movement-related visual (MT, MST, FST) and inferior parietal cortical regions (e.g. PGi and PGs), even though the stimuli are stationary. Tools activate an intermediate visual ventral cortical stream (VMV3, VVC, PHA3) medial to the FFC and also movement-related regions FST and inferior parietal cortex (PGs, PFm). These selective activations to different types of visual stimuli provide important evidence about the functions of cortical regions whose effective and functional connectivity maps have been elucidated recently (Rolls et al. 2023b, 2023e; Rolls et al. 2023a, b, c, d, e, f, g, h; Rolls 2024a). It is important to note that all the visual stimuli were stationary, not moving, even though very interestingly some stimuli activated motion-sensitive brain regions.

Combining the evidence presented here from the activations (Fig. 2a) and functional connectivities (Figs. 4 and S4) of cortical regions to faces and the functional and effective connectivities measured in the resting state complemented by diffusion tractography (Rolls et al. 2023b; Rolls 2024a) leads to the identification of several processing streams and cortical regions each performing different computations with face stimuli.

One ventrolateral visual cortical pathway is via V1–V4–V8 and FFC, which can then introduce face information via lateral parahippocampal cortex TF and perirhinal cortex to the hippocampal memory system (Fig. 2) (Rolls et al. 2022b, 2023b; Rolls 2024a). Of course FFC is a large region, which contains not only face patches (Kanwisher et al. 1997; Weiner et al. 2017; Pitcher et al. 2019; Kosakowski et al. 2022); but also object patches that project forward into the inferior temporal visual cortical areas involved in invariant visual object recognition (Grill-Spector et al. 2006; Rolls 2021a, b; Rolls et al. 2023b); and ideograms (or logograms) of words are represented just lateral to faces in the visual word form area in the fusiform gyrus (Dehaene et al. 2005; Dehaene and Cohen 2011; Caffarra et al. 2021; Yeatman and White 2021). Given that scenes are represented medial to the FFC in the parahippocampal gyrus PHA regions (see below), there is a gradient of decreasing size of what is represented from medial to lateral (see further Malach et al. 2002; Kravitz et al. 2011, 2013; Hori et al. 2021; Rolls 2024a).

Another face pathway is to the cortex in the superior temporal sulcus (Fig. S2) [STS visual/semantic stream (Rolls 2024a)], and interestingly these regions have high FC with both the ventral and dorsal visual streams (Figs. 4 and S4). The cortex in the STS is where we discovered neurons in macaques that respond to face expression and to head movements important in making and breaking social contact, and proposed that this was a separate (third) visual pathway important in decoding socially relevant stimuli and that combined information from the ventral and dorsal visual streams to perform this (Baylis et al. 1987; Hasselmo et al. 1989a, b; Hasselmo et al. 1989a, b; Rolls 2024a). That view has been borne out by subsequent evidence, including fMRI in humans (Weiner and Grill-Spector 2015; Pitcher et al. 2019; Deen et al. 2020; Pitcher and Ungerleider 2021), and disconnection of this system from the orbitofrontal cortex has been related to social symptoms in patients with autism spectrum disorders (Cheng et al. 2015, 2016). However, also of interest is that faces activated the visual inferior parietal cortex areas (e.g. PGi, PFm) in which face and object motion for action may be represented (Rolls et al. 2023e). Further, the sight of stationary faces also activated mainly somatosensory regions of the inferior parietal cortex such as PF, and even also somatosensory regions (Fig. 2, and this was reflected in the FC too in Fig. S3), which may relate to face attributes such as the smoothness of skin.

For scenes (termed places), the selective activations are much more medial than for faces, in the parahippocampal regions PHA1-3 and ventromedial visual regions VMV1-3 and VVC. This usefully shows that in the HCP-MMP atlas, these regions are where the parahippocampal scene area [PSA (Rolls 2023d, a) sometimes termed the parahippocampal place area PPA (Epstein and Kanwisher 1998; Epstein 2005, 2008; Epstein and Julian 2013; Kamps et al. 2016; Epstein and Baker 2019; Sulpizio et al. 2020; Natu et al. 2021)] is located, and importantly extends a previous study (Sulpizio et al. 2020) with a smaller sample size than 956 participants and without the benefit of faces, body parts and tools as comparison stimuli to show selectivity. The sample size of 956 participants, the way in which selectivity was measured by comparison with other visual stimuli, and the use of activations shown in parcellated regions rather than a few peaks reported in MNI space, make the present investigation a very strong analysis of cortical regions selective for scenes in humans. We believe that the representation of scenes in these regions is provided by spatial view cells discovered in macaques in the hippocampus and parahippocampal cortex that respond to the part of the scene where a macaque is looking, and code for that in allocentric, world-based coordinates that are relatively independent of eye position, head direction, facing direction in the environment, and place where the individual is located (Rolls et al. 1989, 1998, 1997; Rolls and O'Mara 1995; Robertson et al. 1998; Georges-François et al. 1999; Rolls 2023d, a). Neurons with many similar properties that respond to locations “out there” being viewed in space have been reported in macaques and other primates by others (Wirth et al. 2017; Mao et al. 2021; Yang et al. 2023; Zhu et al. 2023; Piza et al. 2024), and in humans (Ekstrom et al. 2003; Tsitsiklis et al. 2020; Donoghue et al. 2023). In addition, some hippocampal neurons have been recorded in humans that respond during navigation towards the location of a particular goal in a virtual environment (Qasim et al. 2019; Tsitsiklis et al. 2020; Qasim et al. 2021). The functional connectivities selectively related to places (scenes) in a task include connectivities both with earlier ventral visual stream and with dorsal stream/MT + regions, and onwards with hippocampal system regions (Figs. 5 and S5), thereby very usefully complementing in a task what was described previously for resting state functional and effective connectivity (Rolls et al. 2022b, 2023b). Given this neurophysiological evidence, and the connectivity just summarized, it is proposed that the Parahippocampal Scene Area is a route via which hippocampal spatial view cells receive their information about and selectivity for locations in scenes, and are able then to combine this spatial scene information with ‘What’ and Reward information to form episodic memories (Rolls 2023a, c, 2024a, b; Rolls and Treves 2024). The scene stimuli also selectively activated regions ProS (prostriate) and DVT (dorsal visual transitional), which is where the retrosplenial scene area is located (Sulpizio et al. 2020). Interestingly, the scene stimuli also activated the medial orbitofrontal cortex where rewards and pleasant stimuli are represented (Rolls 2019a, b, 2023b; Zhang et al. 2024), and this may be related to the scenes being more pleasant than some of the other stimuli such as the body parts, which selectively activated the lateral orbitofrontal cortex where aversive stimuli are represented (Rolls 2023b).

For the sight of (stationary) body parts, the selective activations included FFC, and reached TE1p and TE2p (Figs. 2c and S2 panel C), which are the last mainly unimodal inferior temporal cortex visual cortical regions (Rolls et al. 2023a, b, c, d, e, f, g, h). However, visual motion cortical regions such as MT, MST, FST and PH, and intraparietal visuomotor action regions AIP and LIPd, were also activated, which is interesting as body parts do often move, even though these visual stimuli were stationary. Semantic language related regions such as temporo-parietal junction TPOJ1, TPOJ2 and TPOJ3 (Rolls et al. 2022a) were also activated. No selective activation of STS regions was found, perhaps because the stimuli were stationary, but the functional connectivities of STS cortical regions with earlier ventral visual stream and dorsal visual stream regions was increased (Figs. 6 and S6). These STS regions are of interest, for in macaques body parts do activate some neurons in these regions (Perrett et al. 1985; Baylis et al. 1987), and the connectivity in humans with ventral and dorsal stream motion regions supports the concept that these STS regions are involved in the perception of moving body parts. In addition, the STS regions had some high functional connectivities with inferior parietal areas, and indeed activations were also found in visual inferior parietal PGi, PGs and PFm, and with mainly somatosensory inferior parietal PF (Fig. 2c) (Rolls et al. 2023e). The activations of the inferior parietal regions by body parts is of great interest, for this provides function-related support for the concept considered in the analysis of the effective and functional connectivity of these inferior parietal regions with anterior temporal lobe semantic regions that the inferior parietal regions are part of a semantic and action-based system for actions in space, which body parts are involved in (Rolls et al. 2023e).

The cortical regions activated by the sight of (stationary) tools also include inferior parietal cortex regions such as PGs and PFm (Fig. 2d; in the right hemisphere Fig. S2 panel D), and it is proposed for the same reason as above, that tools engage inferior parietal HCP-MMP division regions involved in actions in space (Rolls et al. 2023e), as well as visual motion/visuomotor regions such as FST, LIPv and 7PC. Interestingly, there is also some activation of auditory cortical regions A4 and A5, consistent with raising activity in these regions to the sight of tools because it is a property of tools that they are typically noisy. Consistent with that concept, the sight of tools also activates somatosensory regions such as 1 and 3a, and OP2-3 (Rolls et al. 2023d). These concepts of how the properties of seen stimuli are reflected in different cortical regions involved in different types of processing is consistent with what has been reported with auditory (speech) stimuli (Huth et al. 2016), but the present results reveal that brain regions that process different properties of objects (such as their motion, actions that can be performed with them, feel, and sound) can be activated by purely visual stimuli that are stationary. Again, this function-related evidence is an important complement to the connectivity maps made based on resting state fMRI (Rolls et al. 2023b; Rolls et al. 2023d, e). The sight of tools also activates ventromedial visual stream regions such as V8, VMV3, VMV2, VVC and PHA3 which are medial to the FFC visual face and object regions, and lateral compared to the regions activated by scenes (Rolls 2024a). Tool representations are sandwiched between face and object regions laterally, and scene-related regions medially (Fig. S2 for the right hemisphere). The FCs related to the sight of tools are consistent with these concepts, with for example selectively high FCs when viewing tools between the lateral VMV regions (VMV3 and VVC) and inferior parietal and superior parietal HCP-MMP division regions (Fig. S7).

In terms of topology, a size gradient from large visual stimuli medially in the temporal lobe to small visual stimuli laterally was evident in these analyses. The scene-activated regions were medially in VMV1-3, VVC, and PHA1-3 (Figs. 2 and S2). The tools activated regions more laterally than scenes, including VMV3, VVC and PHA3. The faces activated the FFC more laterally (Figs. 2 and S2). And it is known that the visual word form area (for very small stimuli) is at the lateral border of the FFC (Vinckier et al. 2007). The topology found in this very large sample with the same participants tested with all four types of stimuli complements earlier investigations (Malach et al. 2002; Kravitz et al. 2011, 2013; Hori et al. 2021).

When the effects of increasing the memory load for these visual stimuli from 0-back to 2-back are considered, it is found that activations increase mainly in cortical regions other than those in which these stimuli are represented. The regions that are recruited include especially prefrontal cortex regions implicated in working memory, but also for all stimulus types more activations are found in some inferior parietal cortex regions (especially PFm), and in some cases posterior cingulate division and other cortical regions (Fig. 8). The recruitment of prefrontal cortex regions is consistent with the fact that this is a short-term or working memory task, in which the prefrontal cortex is involved (Goldman-Rakic 1996; Deco et al. 2010; Miller 2013; Fuster 2015; Lundqvist et al. 2018; Miller et al. 2018), rather than a hippocampal episodic memory task (Rolls 2023c; Rolls and Treves 2024). However, as reported in the results, it was found that the parahippocampal cortical regions (PHA and VMV) were selectively activated by scenes in both the right and left hemispheres; and that the hippocampus, entorhinal cortex, perirhinal cortex and lateral parahippocampal cortex TF were activated more by visual stimuli of body parts on the left, and by faces on the right (Tables S3 and S5).

The selective activations and increases in functional connectivity revealed here to stationary visual stimuli have interesting implications for understanding cortical function in humans, apart from the selectivity discussed above.

First, although the visual stimuli were stationary, some of the stimuli activated cortical regions that are especially involved in visual motion, and this was produced by some stimulus types more than others. In particular, stationary body parts activated visual motion MT + complex regions LO1-3, V4t, MT, MT, FST and PH; and stationary tools activated LO1, LO2, FST and PH (Figs. 2 and S2). Both types of stimuli in the natural environment are typically associated with visual motion, and this association perhaps acting through other cortical regions results in visual motion regions coming into activity to influence the representation of these types of stimuli, by bringing in visual motion cortical regions. In the case of faces, most of the STS cortical regions become involved in representing the motion of faces and heads, and to some extent other body parts, by having inputs from both ventral and dorsal cortical visual streams.

Second, some cortical regions involved in processing in other sensory modalities were activated by some of these visual stimulus types. For example, the sight of stationary tools activated auditory cortical regions A4 and A5; and somatomotor cortical regions 1, 2, OP2-3 and 4; and the sight of faces activated somatosensory regions 1 and 2, and inferior parietal regions PF, PFt, PFop with mainly somatosensory inputs (Rolls et al. 2023d, e) (Figs. 2 and S2). The use of tools in the natural environment is typically noisy and involves somatomotor operations, and both are attributes of tools that are raised in the neural representation when humans just look at a stationary tool. It is as if the sound, touch and movement attributes of stationary visual stimuli raise activity in the cortical regions associated with these types of information processing. This speaks to the nature of semantic representations in the human cerebral cortex, which in this case with a unimodal visual stimulus are associated with activations in other modality-specific regions. This is a little different from the situation in which humans listened to hours of narrative stories, and cortical regions appropriate for what was in a story were activated (Huth et al. 2016). In that case, one starts with a semantic (word-level) representation, and finds that cortical regions such as visual motion areas might be activated more by some types of narrative than by others. Here we show that an input in a particular sensory modality, vision, can, depending on the type of visual stimulus, activate cortical regions in other sensory modalities. The mechanism might of course be via semantic cortical regions, as considered next.

Third, some language/semantic cortical regions [identified by meta-analysis (Milton et al. 2021) and effective connectivity (Rolls et al. 2022a)] are activated by these unimodal visual stimuli. While this is not surprising, it was especially evident for faces (which activated TPOJ1-2, STV, PSL, temporal pole TGd, and STS regions, Figs. 2 and S2); and body parts (which activated TPOJ1-3 and TGd especially in the left hemisphere). This is the opposite, bottom-up, direction to the top-down semantic effects investigated by Huth et al (2016).

Fourth, it is of interest that relative to the other stimuli, viewing spatial scenes, and to a lesser extent tools, activated the reward-related medial orbitofrontal cortex. This may be in part because the contrast is with all of the stimuli in Fig. 2, and the sight of body parts selectively activated the aversive-related lateral orbitofrontal cortex (Figs. 2, S2) (Rolls 2023b; Zhang et al. 2024). The implication is that even when performing a 0-back memory task, the reward and punishment, i.e. emotion-related, associations of different types of visual stimuli are being decoded and represented in the emotional systems of the brain (Fig. 3 panel C) (Rolls 2023b; Zhang et al. 2024). However, these orbitofrontal cortex effects were evident when the comparison was with other stimuli, and were not evident in the post-stimulus—the pre-stimulus contrasts shown in Fig. 3.

A possible limitation was that the baseline used for the selective activations shown in Fig. 2 was the mean of the activation to the other four stimuli. This might have meant that the activations to one stimulus type reflected the activations to the other three stimulus types, and indeed that was part of the concept, for the aim of the research described here was to show where activations are selective for the different types of stimuli used here, faces, places, tools, and body parts, rather than to show that all activate for example the primary visual cortex. However, to show the activations for each type of stimulus that were not related to other stimulus types, Fig. 3 shows an analysis in which the activation to each stimulus type (e.g. scenes) was compared to the prestimulus baseline, which enables activation to e.g. scenes to be shown with no effect related to other stimuli. Figure 3 confirms the selectivity of the high order cortical regions activated by each stimulus type. For example, faces produce high activations in FFC, lateral parahippocampal TF, and inferior temporal cortex TE1p and TE2p, and superior temporal sulcus regions STSdp and STSda; and also produce activations in somatosensory 3b, 1 and 2 (Fig. 3a). Continuing, places (scenes) produce high activations in ventromedial visual cortical regions VMV3, VMV2, and VVC, and in medial parahippocampal PHA3, PHA1 and PHA2 (Fig. 3b). Body parts produce high activations in FFC, TF, TE1p and TE2p, but also in STSdp, STSvp; the regions just posterior to this in the temporo-parieto-occipital junction TPOJ2, TPOJ3 and TPOJ1; and also in movement-related visual cortical regions MT, MST, FST; in inferior parietal visual action regions including PGi and PGs; and in posterior cingulate division regions (Fig. 3c). Tools produce activations a little more medial to faces in VVC and VMV3; and in FST; and in somatosensory 1 and 2 (Fig. 3d). Thus the key analyses of selective activations shown in Fig. 2 and elsewhere are supported by the analyses of post-stimulus compared to pre-stimulus activations shown in Fig. 3. The results shown in Fig. 2 (and in Fig. S3C) are also supported by an analysis in which the baseline for each stimulus type was the mean of the activations to the other three stimulus types (Fig. S3D), a type of baseline that has been used previously (Grill-Spector et al. 1998; Stigliani et al. 2015; Natu et al. 2019; Nordt et al. 2021). Another feature of what is shown in Fig. 2 and in many other figures is that the selective activations to each of faces, scenes, body parts, and tools are not single peaks at specified MNI coordinates, but extend over a number of different cortical regions, not only within a specific modality such as visual cortical regions, but also extending to cortical regions involved in processing in other modalities, such as the somatosensory cortex, based on activations measured in 956 Human Connectome Project participants and expressed in the HCP-MMP atlas (Glasser et al. 2016a).

## Conclusions

New and key findings include the following. First, we showed the selective activations (against a mean baseline across all four stimulus types) to stationary images of faces, scenes, body parts, and tools provided for all 360 regions in the HCP-MMP atlas with 956 participants. This provides the largest analysis we know of for example scene areas in the human brain when compared to activations with a range of other visual stimulus types.

Second, because we analysed the activations present in every HCP-MMP cortical region, we were able to go beyond describing the activation to a class of stimulus by one or several peaks identified by MNI coordinates. Instead, we demonstrated the extent to which the cortical activations can in a graded way be found in a number of cortical regions, which moreover can extend beyond classical visual cortical regions to semantically related cortical regions such as somatosensory and auditory and orbitofrontal cortex regions depending on the type of the visual stimulus.

Third, we were able to analyse across the whole cortex with 360 cortical regions the selectively high functional connectivities to images of faces, scenes, body parts, and tools provided for all 360 regions in the HCP-MMP atlas with 956 participants, to show how the connectivity between different cortical regions changes when the processing is changed by different types of visual stimuli.

Fourth, we were able to identify cortical regions and pathways that transmit information beyond primarily visual cortical regions to the hippocampal memory system for different types of stimuli (e.g. scenes vs. faces and objects), which is a topic of great current interest given that the evidence here is on humans, a primate in which the visual representation is of scenes more than of places, whereas in rodents hippocampal representations are mainly about places (O'Keefe 1979; Burgess and O'Keefe 1996; Moser et al. 2017; Rolls 2023a). Indeed, understanding the pathways that provide for the spatial view responses of neurons in the primate including human parahippocampal cortex and hippocampus is part of a revolution in our understanding of hippocampal function in primates and humans, for the proposal is that spatial ‘Where’ representations of scenes in primates including humans are built by feature combinations in a ventromedial visual cortical stream, not in the parietal cortex (Rolls 2023a, 2024; Rolls et al. 2023b; Rolls and Treves 2024; Rolls et al. 2024a, b).

Fifth, we were able to show here that even stationary visual stimuli activate visual motion regions of the human cortex depending on the extent to which the stimuli (e.g. tools) imply motion, compared to other visual stimuli (e.g. scenes) that do not.

Finally, we emphasise that in order to understand better the computational functions of each cortical region, it is important to be able to combine not only cortical connectivity maps using the HCP-MMP parcellation (Glasser et al. 2016a), as previously investigated with resting state fMRI analyses of effective and functional connectivity and with diffusion tractography (Rolls et al. 2022a, 2022b, 2023a, 2023b; Rolls 2023c, 2024; Rolls et al. 2023a, b, 2023c; d, e; Rolls et al. 2023a, b, c, d, e, f, g, h), but to add function to each of the 360 cortical regions in the HCP-MMP atlas, which the present investigation does add for many cortical regions by measuring selective task-related cortical activations and functional connectivities to faces, scenes, body parts, and tools.

### Supplementary Information

Below is the link to the electronic supplementary material.

Supplementary file 1 (PDF 33093 kb)

## Data Availability

The data are available at the HCP website http://www.humanconnectome.org/. Standard Matlab functions were used to calculate the functional connectivity, to perform the paired t-test analyses, and to perform the Bonferroni and FDR corrections for multiple comparisons.
