# Supplementary material for: Selective activations and functional connectivities to the sight of faces, scenes, body parts and tools in visual and non-visual cortical regions leading to the human hippocampus
Source: Brain Struct Funct. 2024 Jun 5;229(6):1471–93. doi: 10.1007/s00429-024-02811-6 (PMC11176242; doi:10.1007/s00429-024-02811-6)
Supplement: Supplementary file 1 — (PDF 33093 kb) [file 429_2024_2811_MOESM1_ESM.pdf]

**Selective activations and functional connectivities to the sight of faces, scenes, body parts and tools in visual and non-visual cortical regions leading to the human hippocampus**

Brain Structure and Function (2024) doi: 10.1007/s00429-024-02811-6

**Supplementary Material**

Edmund T Rolls[1,2,3,*], Jianfeng Feng[1,2] and Ruohan Zhang[1,*]

1. Department of Computer Science, University of Warwick, Coventry, CV4 7AL, UK
2. Institute of Science and Technology for Brain Inspired Intelligence, Fudan University, Shanghai 200403, China
3. Oxford Centre for Computational Neuroscience, Oxford, UK

*Corresponding and co-first author information:

Professor Edmund T. Rolls,

Department of Computer Science, University of Warwick, Coventry CV4 7AL, UK.

Email: Edmund.Rolls@oxcns.org URL: https://www.oxcns.org

https://orcid.org/0000-0003-3025-1292

Ms Ruohan Zhang

Department of Computer Science, University of Warwick, Coventry CV4 7AL, UK.

Email: Ruohan.Zhang.1@warwick.ac.uk

## Modified ordering of the HCP-MMP atlas

The atlas used to define brain regions was the HCP-MMP surface-based atlas (Glasser et al. 2016), illustrated in Figs. S1. In the HCP-MMP atlas, each region has its RegionID, which we show in Table S1. Detailed information about the regions is available in the Supplementary Material File NIHMS68870-supplement-Neuroanatomical_Supplementary_Results.pdf provided by Glasser et al (2016). In that Supplementary Material file, a grouping of the regions is suggested based on geographic proximity and functional similarities, and this grouping is shown in the column labelled CortexID in Table S1. That has led to a different ordering of the regions, which we show in Table S1, with the original regionIDs from the HCP atlas shown in the column headed 'regionID'. This reordered version of the HCP-MMP atlas is described by Dr Dianne Patterson of the University of Arizona at https://neuroimaging-core-docs.readthedocs.io/en/latest/pages/atlases.html, where the following supporting files used to help generate Table S1 are available: HCP-MMP_UniqueRegionList.csv and Glasser_2016_Table.xlsx. We made file HCPMMP_CortexID_Ordering.xlsx from this, and this is available with the HCex (Huang, Rolls, Feng and Lin 2022). The connectivity matrices shown in the present paper used the ordering shown in Table S1, which is also used in the volumetric and extended form of this atlas, HCPex (Huang, Rolls, Feng and Lin 2022).

**Table S1.** Regions defined in the modified Human Connectome Project atlas (Glasser et al. 2016). L=left hemisphere, R=right. The column 'Reordered region ID' is that used in Figs. 4-7, and is a reordering of that based on suggestions in the Supplementary Information of Glasser et al (2016). In that Supplementary Information of that paper, the 360 regions are grouped and ordered based on geographic proximity and functional similarities as shown in column 'Original ID. The regions were reordered and reorganized by Dr Dianne Patterson of the University of Arizona at and https://neuroimaging-core-docs.readthedocs.io/en/latest/pages/atlases.html with the HCP-MMP_UniqueRegionList.csv, and are shown in the column labelled CortexID in Table S1 which is the order used in HCPex atlas (Huang, Rolls, Feng and Lin 2022) and in this paper. The volumes are in $mm^3$. This modified HCPex atlas with the reordering is described elsewhere (Huang, Rolls, Feng and Lin 2022). All the analyses described in this paper were performed for the surface-based HCP-MMP registration..

| Reordered ID (L, R) | Region | RegionLongName | Cortical Division | Cortex ID | Original ID | Voxel numbers ($1mm^3$) (L,R) |
|---|---|---|---|---|---|---|
| 1, 181 | V1 | Primary_Visual_Cortex | Primary_Visual | 1 | 1 | 13812, 13406 |
| 2, 182 | V2 | Second_Visual_Area | Early_Visual | 2 | 4 | 9515, 9420 |
| 3, 183 | V3 | Third_Visual_Area | Early_Visual | 2 | 5 | 7106, 7481 |
| 4, 184 | V4 | Fourth_Visual_Area | Early_Visual | 2 | 6 | 4782, 4537 |
| 5, 185 | IPS1 | IntraParietal_Sulcus_Area_1 | Dorsal_Stream_Visual | 3 | 17 | 1751, 1750 |
| 6, 186 | V3A | Area_V3A | Dorsal_Stream_Visual | 3 | 13 | 2191, 2212 |
| 7, 187 | V3B | Area_V3B | Dorsal_Stream_Visual | 3 | 19 | 639, 731 |
| 8, 188 | V6 | Sixth_Visual_Area | Dorsal_Stream_Visual | 3 | 3 | 1402, 1559 |
| 9, 189 | V6A | Area_V6A | Dorsal_Stream_Visual | 3 | 152 | 904, 734 |
| 10, 190 | V7 | Seventh_Visual_Area | Dorsal_Stream_Visual | 3 | 16 | 1005, 1041 |
| 11, 191 | FFC | Fusiform_Face_Complex | Ventral_Stream_Visual | 4 | 18 | 3848, 4402 |
| 12, 192 | PIT | Posterior_InferoTemporal_complex | Ventral_Stream_Visual | 4 | 22 | 1392, 1386 |
| 13, 193 | V8 | Eighth_Visual_Area | Ventral_Stream_Visual | 4 | 7 | 1361, 1175 |
| 14, 194 | VMV1 | VentroMedial_Visual_Area_1 | Ventral_Stream_Visual | 4 | 153 | 939, 1219 |
| 15, 195 | VMV2 | VentroMedial_Visual_Area_2 | Ventral_Stream_Visual | 4 | 160 | 639, 923 |
| 16, 196 | VMV3 | VentroMedial_Visual_Area_3 | Ventral_Stream_Visual | 4 | 154 | 941, 1242 |
| 17, 197 | VVC | Ventral_Visual_Complex | Ventral_Stream_Visual | 4 | 163 | 2487, 2753 |
| 18, 198 | FST | Area_FST | MT+_Complex | 5 | 157 | 1324, 1683 |
| 19, 199 | LO1 | Area_Lateral_Occipital_1 | MT+_Complex | 5 | 20 | 619, 909 |
| 20, 200 | LO2 | Area_Lateral_Occipital_2 | MT+_Complex | 5 | 21 | 1179, 1062 |
| 21, 201 | LO3 | Area_Lateral_Occipital_3 | MT+_Complex | 5 | 159 | 438, 915 |
| 22, 202 | MST | Medial_Superior_Temporal_Area | MT+_Complex | 5 | 2 | 794, 1036 |
| 23, 203 | MT | Middle_Temporal_Area | MT+_Complex | 5 | 23 | 620, 1005 |

| | | | | | | |
|---|---|---|---|---|---|---|
| 24, 204 | PH | Area_PH | MT+_Complex | 5 | 138 | 3453, 3205 |
| 25, 205 | V3CD | Area_V3CD | MT+_Complex | 5 | 158 | 876, 1222 |
| 26, 206 | V4t | Area_V4t | MT+_Complex | 5 | 156 | 1037, 1249 |
| 27, 207 | 1 | Area_1 | SomaSens_Motor | 6 | 51 | 6590, 5925 |
| 28, 208 | 2 | Area_2 | SomaSens_Motor | 6 | 52 | 4278, 4727 |
| 29, 209 | 3a | Area_3a | SomaSens_Motor | 6 | 53 | 2247, 2286 |
| 30, 210 | 3b | Primary_Sensory_Cortex | SomaSens_Motor | 6 | 9 | 5451, 4350 |
| 31, 211 | 4 | Primary_Motor_Cortex | SomaSens_Motor | 6 | 8 | 10776, 10254 |
| 32, 212 | 23c | Area_23c | ParaCentral_MidCing | 7 | 38 | 2259, 2498 |
| 33, 213 | 24dd | Dorsal_Area_24d | ParaCentral_MidCing | 7 | 40 | 2665, 2820 |
| 34, 214 | 24dv | Ventral_Area_24d | ParaCentral_MidCing | 7 | 41 | 1076, 1349 |
| 35, 215 | 5L | Area_5L | ParaCentral_MidCing | 7 | 39 | 2249, 2327 |
| 36, 216 | 5m | Area_5m | ParaCentral_MidCing | 7 | 36 | 1483, 2079 |
| 37, 217 | 5mv | Area_5m_ventral | ParaCentral_MidCing | 7 | 37 | 1651, 1996 |
| 38, 218 | 6ma | Area_6m_anterior | ParaCentral_MidCing | 7 | 44 | 3941, 4251 |
| 39, 219 | 6mp | Area_6mp | ParaCentral_MidCing | 7 | 55 | 3701, 3105 |
| 40, 220 | SCEF | Supplementary_and_Cingulate_Eye_ Field | ParaCentral_MidCing | 7 | 43 | 3500, 3371 |
| 41, 221 | 55b | Area_55b | Premotor | 8 | 12 | 2422, 1537 |
| 42, 222 | 6a | Area_6_anterior | Premotor | 8 | 96 | 4233, 3752 |
| 43, 223 | 6d | Dorsal_area_6 | Premotor | 8 | 54 | 2916, 2909 |
| 44, 224 | 6r | Rostral_Area_6 | Premotor | 8 | 78 | 3029, 3981 |
| 45, 225 | 6v | Ventral_Area_6 | Premotor | 8 | 56 | 2075, 2516 |
| 46, 226 | FEF | Frontal_Eye_Fields | Premotor | 8 | 10 | 1787, 1889 |
| 47, 227 | PEF | Premotor_Eye_Field | Premotor | 8 | 11 | 1006, 1258 |
| 48, 228 | 43 | Area_43 | Posterior_Opercular | 9 | 99 | 1889, 1678 |
| 49, 229 | FOP1 | Frontal_Opercular_Area_1 | Posterior_Opercular | 9 | 113 | 879, 932 |
| 50, 230 | OP1 | Area_OP1-SII | Posterior_Opercular | 9 | 101 | 1275, 1072 |
| 51, 231 | OP2-3 | Area_OP2-3-VS | Posterior_Opercular | 9 | 102 | 943, 792 |
| 52, 232 | OP4 | Area_OP4-PV | Posterior_Opercular | 9 | 100 | 2332, 2409 |
| 53, 233 | 52 | Area_52 | Early_Auditory | 10 | 103 | 725, 580 |
| 54, 234 | A1 | Primary_Auditory_Cortex | Early_Auditory | 10 | 24 | 1023, 796 |
| 55, 235 | LBelt | Lateral_Belt_Complex | Early_Auditory | 10 | 174 | 820, 901 |
| 56, 236 | MBelt | Medial_Belt_Complex | Early_Auditory | 10 | 173 | 1242, 1236 |
| 57, 237 | PBelt | ParaBelt_Complex | Early_Auditory | 10 | 124 | 1719, 1439 |
| 58, 238 | PFcm | Area_PFcm | Early_Auditory | 10 | 105 | 1486, 1485 |
| 59, 239 | RI | RetroInsular_Cortex | Early_Auditory | 10 | 104 | 1149, 1334 |

| | | | | | | |
|---|---|---|---|---|---|---|
| 60, 240 | A4 | Auditory_4_Complex | Auditory_Association | 11 | 175 | 3514, 3610 |
| 61, 241 | A5 | Auditory_5_Complex | Auditory_Association | 11 | 125 | 3346, 3881 |
| 62, 242 | STGa | Area_STGa | Auditory_Association | 11 | 123 | 2509, 2187 |
| 63, 243 | STSda | Area_STSd_anterior | Auditory_Association | 11 | 128 | 1944, 2389 |
| 64, 244 | STSdp | Area_STSd_posterior | Auditory_Association | 11 | 129 | 1994, 2605 |
| 65, 245 | STSva | Area_STSv_anterior | Auditory_Association | 11 | 176 | 1694, 1900 |
| 66, 246 | STSvp | Area_STSv_posterior | Auditory_Association | 11 | 130 | 2898, 2515 |
| 67, 247 | TA2 | Area_TA2 | Auditory_Association | 11 | 107 | 1518, 1726 |
| 68, 248 | AAIC | Anterior_Agranular_Insula_Complex | Insula_FrontalOperc | 12 | 112 | 1859, 1691 |
| 69, 249 | AVI | Anterior_Ventral_Insular_Area | Insula_FrontalOperc | 12 | 111 | 1446, 1792 |
| 70, 250 | FOP2 | Frontal_Opercular_Area_2 | Insula_FrontalOperc | 12 | 115 | 750, 720 |
| 71, 251 | FOP3 | Frontal_Opercular_Area_3 | Insula_FrontalOperc | 12 | 114 | 754, 614 |
| 72, 252 | FOP4 | Frontal_Opercular_Area_4 | Insula_FrontalOperc | 12 | 108 | 2522, 1678 |
| 73, 253 | FOP5 | Area_Frontal_Opercular_5 | Insula_FrontalOperc | 12 | 169 | 1297, 1365 |
| 74, 254 | Ig | Insular_Granular_Complex | Insula_FrontalOperc | 12 | 168 | 841, 1077 |
| 75, 255 | MI | Middle_Insular_Area | Insula_FrontalOperc | 12 | 109 | 2102, 1960 |
| 76, 256 | PI | Para-Insular_Area | Insula_FrontalOperc | 12 | 178 | 1033, 1058 |
| 77, 257 | Pir | Piriform_Cortex | Insula_FrontalOperc | 12 | 110 | 2287, 1856 |
| 78, 258 | PoI1 | Area_Posterior_Insular_1 | Insula_FrontalOperc | 12 | 167 | 1811, 1835 |
| 79, 259 | PoI2 | Posterior_Insular_Area_2 | Insula_FrontalOperc | 12 | 106 | 2747, 2675 |
| 80, 260 | H | Hippocampus | Medial_Temporal | 13 | 120 | 4283, 3626 |
| 81, 261 | PreS | PreSubiculum | Medial_Temporal | 13 | 119 | 1817, 1558 |
| 82, 262 | EC | Entorhinal_Cortex | Medial_Temporal | 13 | 118 | 2127, 2110 |
| 83, 263 | PeEc | Perirhinal_Ectorhinal_Cortex | Medial_Temporal | 13 | 122 | 4826, 4755 |
| 84, 264 | TF | Area_TF | Medial_Temporal | 13 | 135 | 3986, 4752 |
| 85, 265 | PHA1 | ParaHippocampal_Area_1 | Medial_Temporal | 13 | 126 | 1281, 1168 |
| 86, 266 | PHA2 | ParaHippocampal_Area_2 | Medial_Temporal | 13 | 155 | 783, 771 |
| 87, 267 | PHA3 | ParaHippocampal_Area_3 | Medial_Temporal | 13 | 127 | 2023, 1122 |
| 88, 268 | PHT | Area_PHT | Lateral_Temporal | 14 | 137 | 4182, 3410 |
| 89, 269 | TE1a | Area_TE1_anterior | Lateral_Temporal | 14 | 132 | 5227, 4180 |
| 90, 270 | TE1m | Area_TE1_Middle | Lateral_Temporal | 14 | 177 | 3339, 3429 |
| 91, 271 | TE1p | Area_TE1_posterior | Lateral_Temporal | 14 | 133 | 7116, 6010 |
| 92, 272 | TE2a | Area_TE2_anterior | Lateral_Temporal | 14 | 134 | 5691, 5753 |
| 93, 273 | TE2p | Area_TE2_posterior | Lateral_Temporal | 14 | 136 | 4115, 3040 |
| 94, 274 | TGd | Area_TG_dorsal | Lateral_Temporal | 14 | 131 | 10192, 10269 |
| 95, 275 | TGv | Area_TG_Ventral | Lateral_Temporal | 14 | 172 | 3694, 4515 |
| 96, 276 | PSL | PeriSylvian_Language_Area | TPO | 15 | 25 | 2154, 2759 |

| | | | | | | |
|---|---|---|---|---|---|---|
| 97, 277 | STV | Superior_Temporal_Visual_Area | TPO | 15 | 28 | 2322, 2294 |
| 98, 278 | TPOJ1 | Area_TemporoParietoOccipital_Juncti on_1 | TPO | 15 | 139 | 2102, 3938 |
| 99, 279 | TPOJ2 | Area_TemporoParietoOccipital_Juncti on_2 | TPO | 15 | 140 | 1930, 2068 |
| 100, 280 | TPOJ3 | Area_TemporoParietoOccipital_Juncti on_3 | TPO | 15 | 141 | 1290, 1277 |
| 101, 281 | 7AL | Lateral_Area_7A | Superior_Parietal | 16 | 42 | 2134, 2030 |
| 102, 282 | 7Am | Medial_Area_7A | Superior_Parietal | 16 | 45 | 2995, 2379 |
| 103, 283 | 7PC | Area_7PC | Superior_Parietal | 16 | 47 | 3151, 3415 |
| 104, 284 | 7PL | Lateral_Area_7P | Superior_Parietal | 16 | 46 | 1695, 1363 |
| 105, 285 | 7Pm | Medial_Area_7P | Superior_Parietal | 16 | 29 | 1601, 1308 |
| 106, 286 | AIP | Anterior_IntraParietal_Area | Superior_Parietal | 16 | 117 | 1999, 2542 |
| 107, 287 | LIPd | Area_Lateral_IntraParietal_dorsal | Superior_Parietal | 16 | 95 | 1008, 869 |
| 108, 288 | LIPv | Area_Lateral_IntraParietal_ventral | Superior_Parietal | 16 | 48 | 1681, 1783 |
| 109, 289 | MIP | Medial_IntraParietal_Area | Superior_Parietal | 16 | 50 | 1872, 2403 |
| 110, 290 | VIP | Ventral_IntraParietal_Complex | Superior_Parietal | 16 | 49 | 1890, 1577 |
| 111, 291 | IP0 | Area_IntraParietal_0 | Inferior_Parietal | 17 | 146 | 1203, 1239 |
| 112, 292 | IP1 | Area_IntraParietal_1 | Inferior_Parietal | 17 | 145 | 1692, 1632 |
| 113, 293 | IP2 | Area_IntraParietal_2 | Inferior_Parietal | 17 | 144 | 2102, 1861 |
| 114, 294 | PF | Area_PF_Complex | Inferior_Parietal | 17 | 148 | 5457, 5251 |
| 115, 295 | PFm | Area_PFm_Complex | Inferior_Parietal | 17 | 149 | 8220, 8141 |
| 116, 296 | PFop | Area_PF_Opercular | Inferior_Parietal | 17 | 147 | 1797, 1783 |
| 117, 297 | PFt | Area_PFt | Inferior_Parietal | 17 | 116 | 1983, 2039 |
| 118, 298 | PGi | Area_PGi | Inferior_Parietal | 17 | 150 | 4791, 4970 |
| 119, 299 | PGp | Area_PGp | Inferior_Parietal | 17 | 143 | 2501, 3740 |
| 120, 300 | PGs | Area_PGs | Inferior_Parietal | 17 | 151 | 4552, 3366 |
| 121, 301 | 23d | Area_23d | Posterior_Cingulate | 18 | 32 | 1261, 1513 |
| 122, 302 | 31a | Area_31a | Posterior_Cingulate | 18 | 162 | 1260, 1116 |
| 123, 303 | 31pd | Area_31pd | Posterior_Cingulate | 18 | 161 | 1428, 864 |
| 124, 304 | 31pv | Area_31p_ventral | Posterior_Cingulate | 18 | 35 | 950, 1022 |
| 125, 305 | 7m | Area_7m | Posterior_Cingulate | 18 | 30 | 2128, 2067 |
| 126, 306 | d23ab | Area_dorsal_23_a+b | Posterior_Cingulate | 18 | 34 | 1607, 1106 |
| 127, 307 | DVT | Dorsal_Transitional_Visual_Area | Posterior_Cingulate | 18 | 142 | 1806, 2176 |
| 128, 308 | PCV | PreCuneus_Visual_Area | Posterior_Cingulate | 18 | 27 | 2245, 2416 |
| 129, 309 | POS1 | Parieto-Occipital_Sulcus_Area_1 | Posterior_Cingulate | 18 | 31 | 2531, 2727 |
| 130, 310 | POS2 | Parieto-Occipital_Sulcus_Area_2 | Posterior_Cingulate | 18 | 15 | 3261, 3093 |

| | | | | | | |
|---|---|---|---|---|---|---|
| 131, 311 | ProS | ProStriate_Area | Posterior_Cingulate | 18 | 121 | 1222, 1055 |
| 132, 312 | RSC | RetroSplenial_Complex | Posterior_Cingulate | 18 | 14 | 2830, 3067 |
| 133, 313 | v23ab | Area_ventral_23_a+b | Posterior_Cingulate | 18 | 33 | 916, 1089 |
| 134, 314 | 10r | Area_10r | AntCing_MedPFC | 19 | 65 | 1589, 1053 |
| 135, 315 | 10v | Area_10v | AntCing_MedPFC | 19 | 88 | 3906, 2667 |
| 136, 316 | 25 | Area_25 | AntCing_MedPFC | 19 | 164 | 1911, 2135 |
| 137, 317 | 33pr | Area_33_prime | AntCing_MedPFC | 19 | 58 | 1354, 1316 |
| 138, 318 | 8BM | Area_8BM | AntCing_MedPFC | 19 | 63 | 3122, 3436 |
| 139, 319 | 9m | Area_9_Middle | AntCing_MedPFC | 19 | 69 | 6338, 5881 |
| 140, 320 | a24 | Area_a24 | AntCing_MedPFC | 19 | 61 | 2085, 2152 |
| 141, 321 | a24pr | Anterior_24_prime | AntCing_MedPFC | 19 | 59 | 1095, 1474 |
| 142, 322 | a32pr | Area_anterior_32_prime | AntCing_MedPFC | 19 | 179 | 1759, 1118 |
| 143, 323 | d32 | Area_dorsal_32 | AntCing_MedPFC | 19 | 62 | 2228, 2374 |
| 144, 324 | p24 | Area_posterior_24 | AntCing_MedPFC | 19 | 180 | 2394, 2442 |
| 145, 325 | p24pr | Area_Posterior_24_prime | AntCing_MedPFC | 19 | 57 | 1422, 1724 |
| 146, 326 | p32 | Area_p32 | AntCing_MedPFC | 19 | 64 | 1180, 1765 |
| 147, 327 | p32pr | Area_p32_prime | AntCing_MedPFC | 19 | 60 | 1569, 1305 |
| 148, 328 | pOFC | Posterior_OFC_Complex | AntCing_MedPFC | 19 | 166 | 2486, 2836 |
| 149, 329 | s32 | Area_s32 | AntCing_MedPFC | 19 | 165 | 604, 1015 |
| 150, 330 | 10d | Area_10d | OrbPolaFrontal | 20 | 72 | 3644, 3096 |
| 151, 331 | 10pp | Polar_10p | OrbPolaFrontal | 20 | 90 | 1997, 2487 |
| 152, 332 | 11l | Area_11l | OrbPolaFrontal | 20 | 91 | 3531, 3793 |
| 153, 333 | 13l | Area_13l | OrbPolaFrontal | 20 | 92 | 2429, 1757 |
| 154, 334 | 47m | Area_47m | OrbPolaFrontal | 20 | 66 | 799, 781 |
| 155, 335 | 47s | Area_47s | OrbPolaFrontal | 20 | 94 | 2795, 3080 |
| 156, 336 | a10p | Area_anterior_10p | OrbPolaFrontal | 20 | 89 | 1964, 1748 |
| 157, 337 | OFC | Orbital_Frontal_Complex | OrbPolaFrontal | 20 | 93 | 4560, 5232 |
| 158, 338 | p10p | Area_posterior_10p | OrbPolaFrontal | 20 | 170 | 2116, 2365 |
| 159, 339 | 44 | Area_44 | Inferior_Frontal | 21 | 74 | 2435, 2589 |
| 160, 340 | 45 | Area_45 | Inferior_Frontal | 21 | 75 | 3762, 2962 |
| 161, 341 | 47l | Area_47l_(47_lateral) | Inferior_Frontal | 21 | 76 | 2527, 2592 |
| 162, 342 | a47r | Area_anterior_47r | Inferior_Frontal | 21 | 77 | 4167, 3763 |
| 163, 343 | IFJa | Area_IFJa | Inferior_Frontal | 21 | 79 | 1513, 1405 |
| 164, 344 | IFJp | Area_IFJp | Inferior_Frontal | 21 | 80 | 960, 740 |
| 165, 345 | IFSa | Area_IFSa | Inferior_Frontal | 21 | 82 | 2057, 2641 |
| 166, 346 | IFSp | Area_IFSp | Inferior_Frontal | 21 | 81 | 1589, 1730 |
| 167, 347 | p47r | Area_posterior_47r | Inferior_Frontal | 21 | 171 | 2133, 1761 |

| | | | | | | |
|---|---|---|---|---|---|---|
| 168, 348 | 46 | Area_46 | Dorsolateral_Prefrontal | 22 | 84 | 4863, 4394 |
| 169, 349 | 8Ad | Area_8Ad | Dorsolateral_Prefrontal | 22 | 68 | 3386, 3492 |
| 170, 350 | 8Av | Area_8Av | Dorsolateral_Prefrontal | 22 | 67 | 4807, 5902 |
| 171, 351 | 8BL | Area_8B_Lateral | Dorsolateral_Prefrontal | 22 | 70 | 3377, 4078 |
| 172, 352 | 8C | Area_8C | Dorsolateral_Prefrontal | 22 | 73 | 4085, 3134 |
| 173, 353 | 9-46d | Area_9-46d | Dorsolateral_Prefrontal | 22 | 86 | 4534, 4666 |
| 174, 354 | 9a | Area_9_anterior | Dorsolateral_Prefrontal | 22 | 87 | 3706, 3048 |
| 175, 355 | 9p | Area_9_Posterior | Dorsolateral_Prefrontal | 22 | 71 | 3426, 2488 |
| 176, 356 | a9-46v | Area_anterior_9-46v | Dorsolateral_Prefrontal | 22 | 85 | 3314, 2628 |
| 177, 357 | i6-8 | Inferior_6-8_Transitional_Area | Dorsolateral_Prefrontal | 22 | 97 | 1764, 2418 |
| 178, 358 | p9-46v | Area_posterior_9-46v | Dorsolateral_Prefrontal | 22 | 83 | 2871, 4635 |
| 179, 359 | s6-8 | Superior_6-8_Transitional_Area | Dorsolateral_Prefrontal | 22 | 98 | 1336, 2132 |
| 180, 360 | SFL | Superior_Frontal_Language_Area | Dorsolateral_Prefrontal | 22 | 26 | 3873, 3055 |

Column 1 (Reordered ID) shows the order in HCPex based on the HCP-MMP1_UniqueRegionList.csv, as described in the Methods, of the 360 cortical regions originally defined by Glasser et al (2016). The names of the cortical divisions shown in column 4 come from the same .csv file. The sixth column shows the original order used by Glasser et al (2016). Abbreviations: L=left hemisphere, R=right. MT+_Complex, MT+_Complex_and_Neighboring_Visual_Areas; SomaSens_Motor, Somatosensory_and_Motor; ParaCentral_MidCing, Paracentral_Lobular_and_Mid_Cingulate; Insula_FrontalOperc, Insular_and_Frontal_Opercular; TPO, Temporo-Parieto-Occipital_Junction; AntCing_MedPFC, Anterior_Cingulate_and_Medial_Prefrontal; OrbPolaFrontal, Orbital_and_Polar_Frontal.

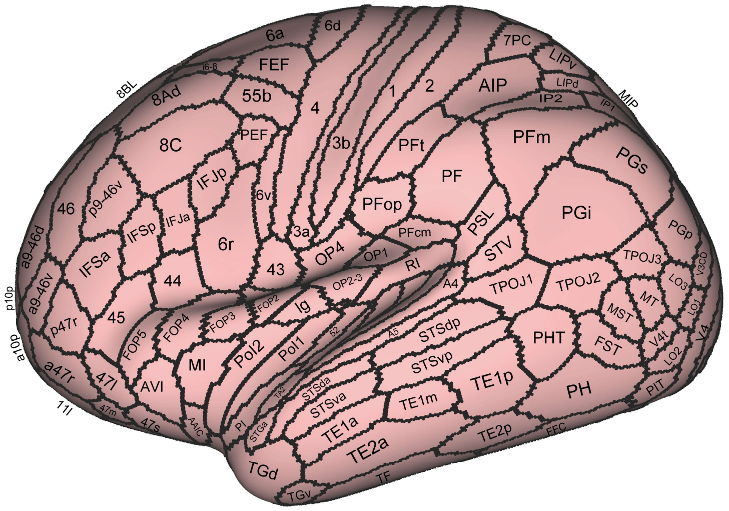


HCP-MMP human brain parcellation: lateral view

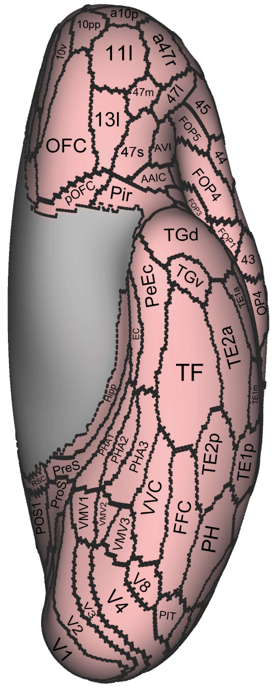


Inferior view

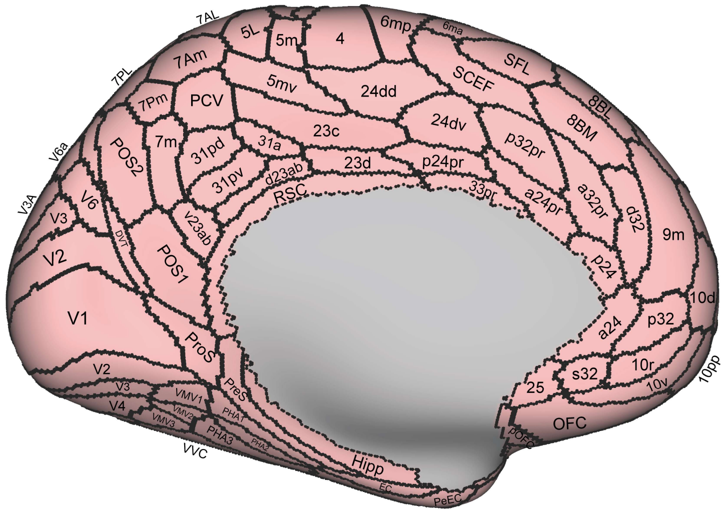


HCP-MMP human brain parcellation: medial view

**Fig. S1. Parcellation of the human cortex** in the HCP-MMP atlas (Glasser, et al., 2016), and in its extended version HCPex (Huang, et al., 2022). The regions are shown on images of the human brain in inflated form with the sulci expanded sufficiently to allow the regions within the sulci to be shown. The abbreviations for each cortical region are provided in Table S1.

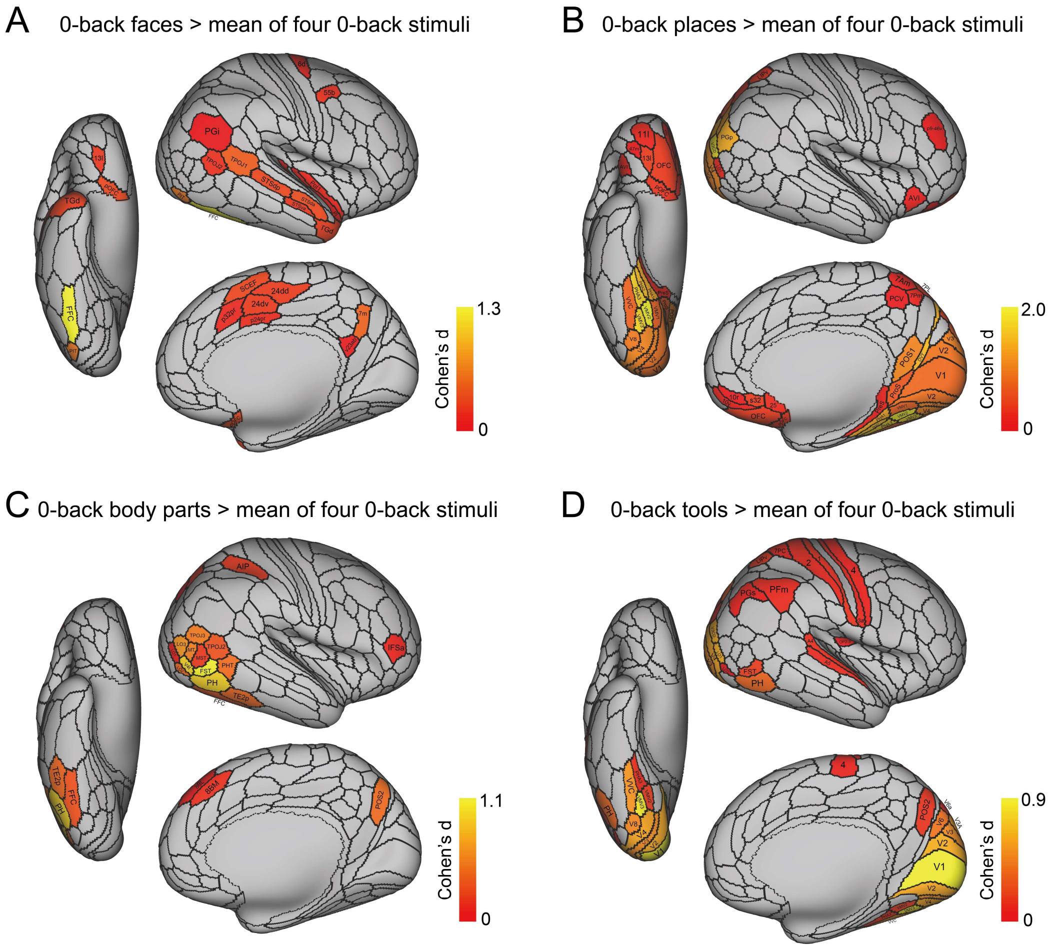


Fig. S2A. Brain regions in the right hemisphere exhibiting significant differences in the average BOLD signal for the four 0-back working memory conditions compared to the mean of these conditions, after Bonferroni correction (α=0.05). Panels (A) and (C) show the top 30% of cortical regions with significant differences for the 0-back faces and 0-back body parts conditions contrasted with the mean of the four conditions, respectively. Panels (B) and (D) display all the brain regions with significant differences for the 0-back places and 0-back tools conditions compared to the mean of these four conditions, respectively.

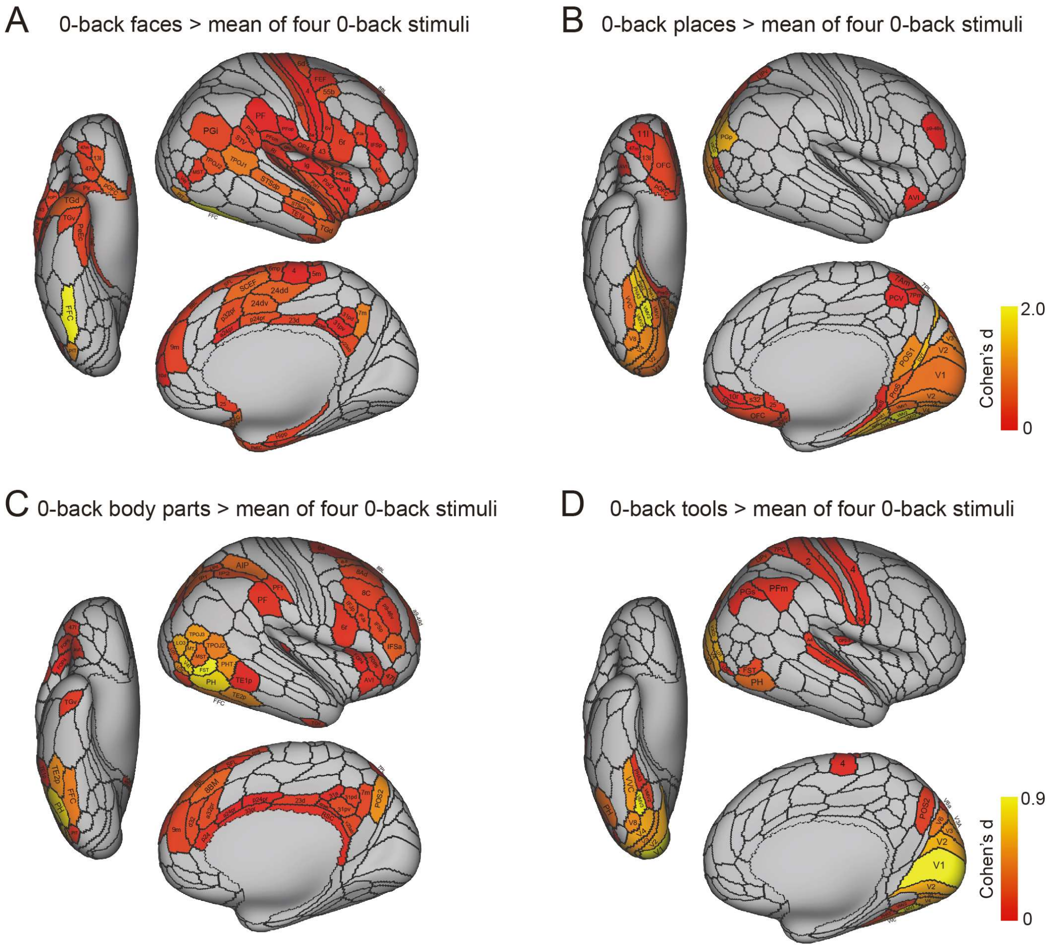


Fig. S2B. Brain regions in the right hemisphere exhibiting significant differences in the average BOLD signal for the four 0-back working memory conditions compared to the mean of these conditions, after Bonferroni correction (α=0.05). This is the same as Fig. S2A, except that all significant activations are included in (A) and (C). (SelectiveActivationsAllRight_label.eps)

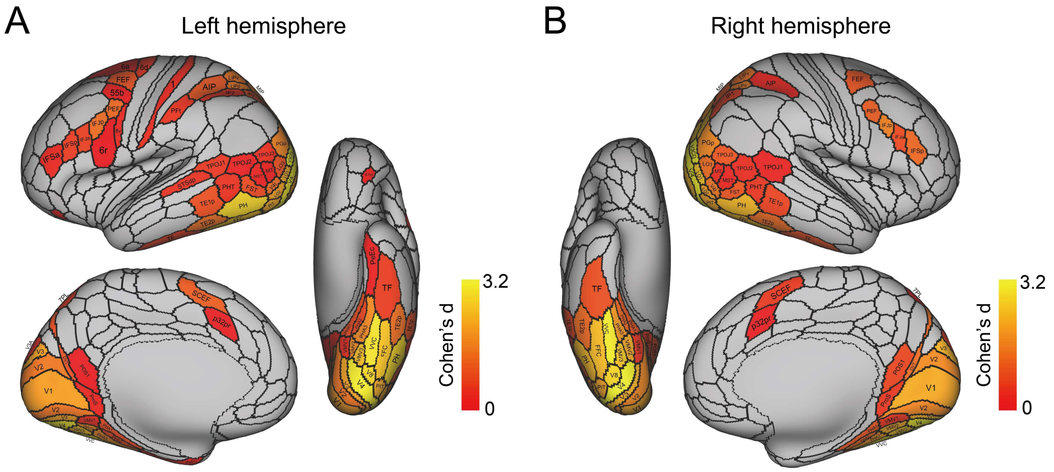


Fig. S3A. The top 50% of cortical regions exhibiting significant differences in the average BOLD signal between the initial 15 timepoints and the last 20 timepoints (when the BOLD signal response to the visual stimuli was occurring) of the mean across four 0-back working memory conditions after Bonferroni correction (α=0.05). Panels (A) and (B) depict brain regions with significant differences in the left and right hemispheres, respectively. The effect size as indicated by Cohen's d is shown.

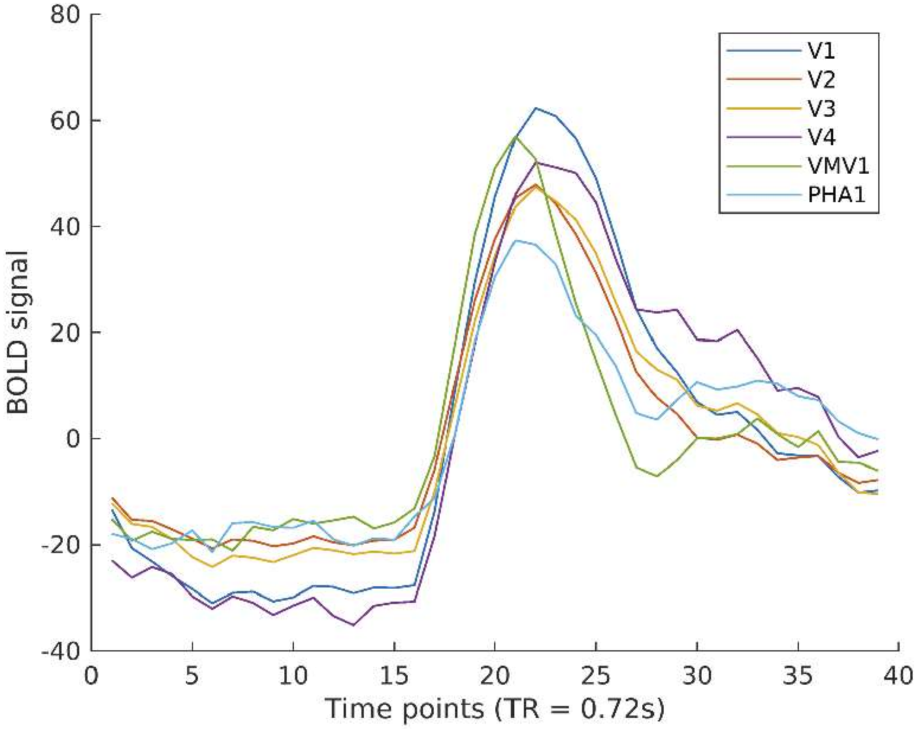


Fig. S3B. The timecourse of the BOLD signal in runs of the 0-back task for different cortical regions and averaged across the four stimulus types of faces, places, body parts, and tools. The cue for the start of a run starts in timebin 1, and the first visual stimulus in the run starts after 2.5 s at timebin 4. (selected_regions_timecourse.eps)

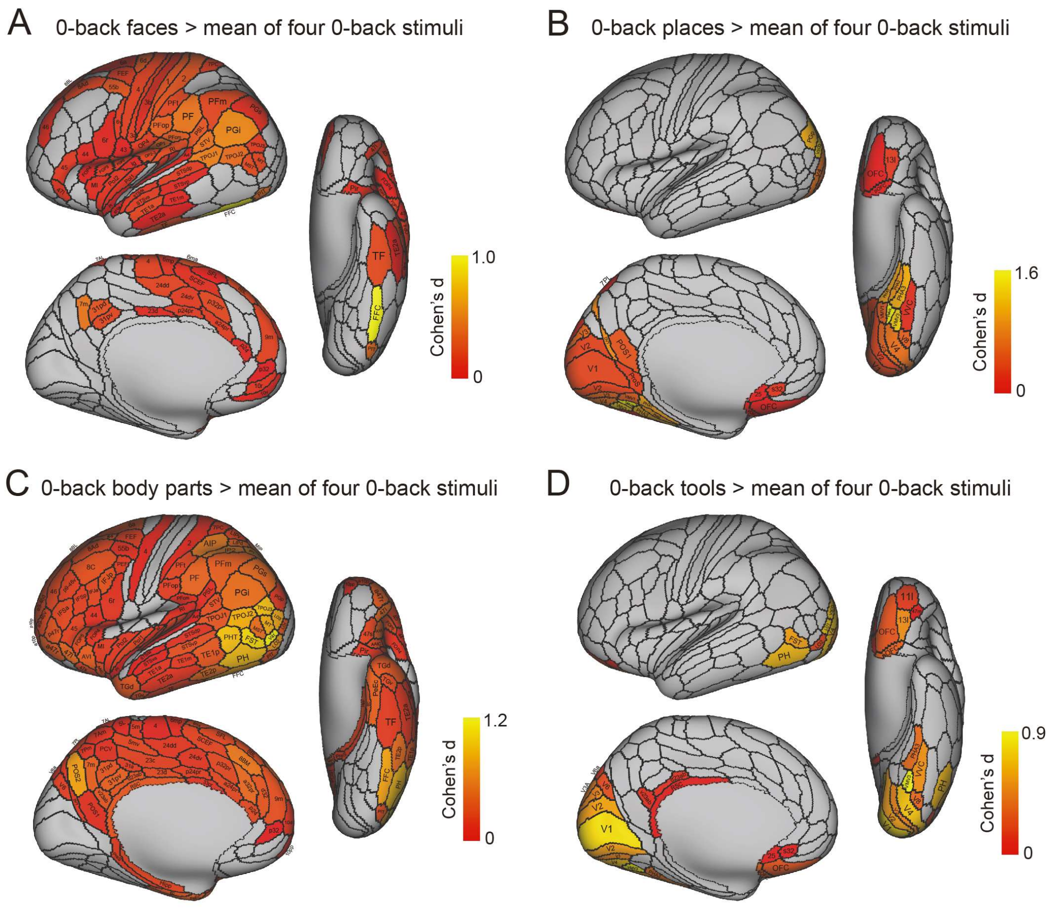


Fig. S3C. Brain regions in the left hemisphere with significant differences in the average BOLD signal for the faces, scenes, body parts, and tools compared to the mean of these conditions in the 0-back working memory task, after Bonferroni correction (α=0.05). This is similar to Fig. 2 of the paper, except that all regions that passed Bonferroni correction are included in (A) and (C). (Fig.S3C.eps)

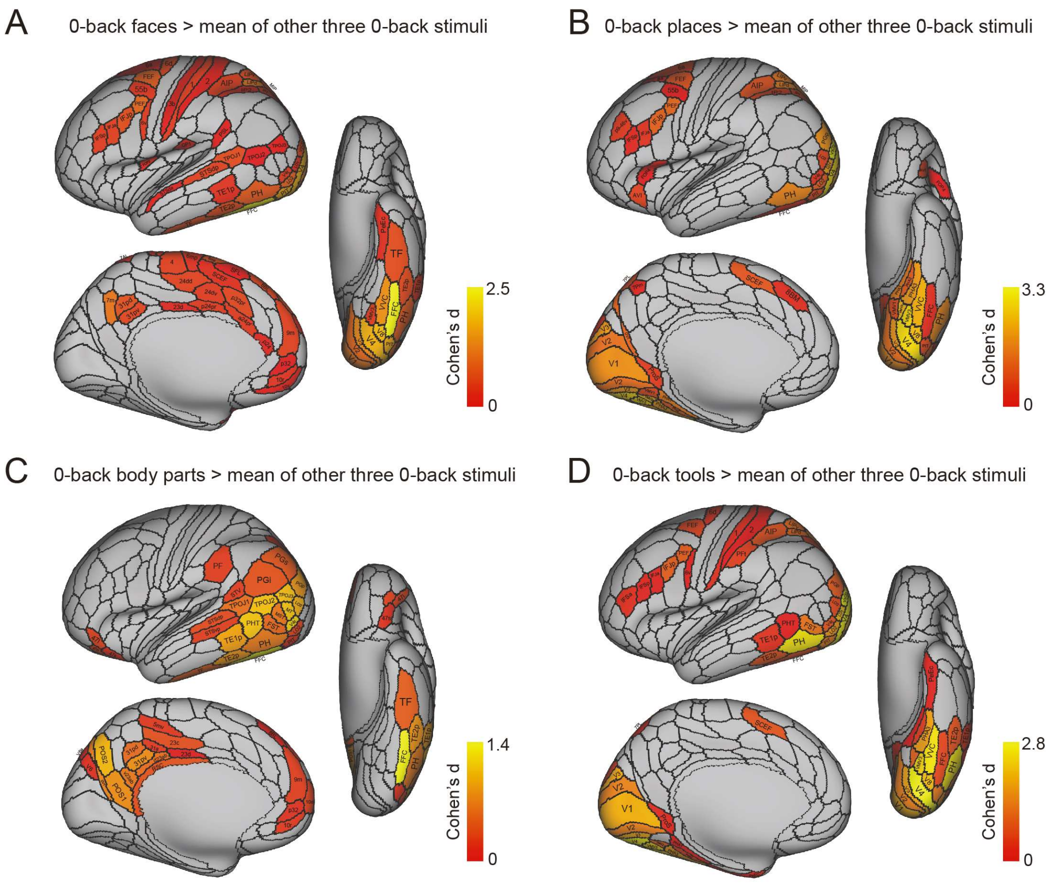


Fig. S3D. Brain regions in the left hemisphere with significant differences in the BOLD signal for the faces, scenes, body parts, and tools compared to the mean of the other three conditions in the 0-back working memory task, after Bonferroni correction (α=0.05). (Fig.S3D.ai)

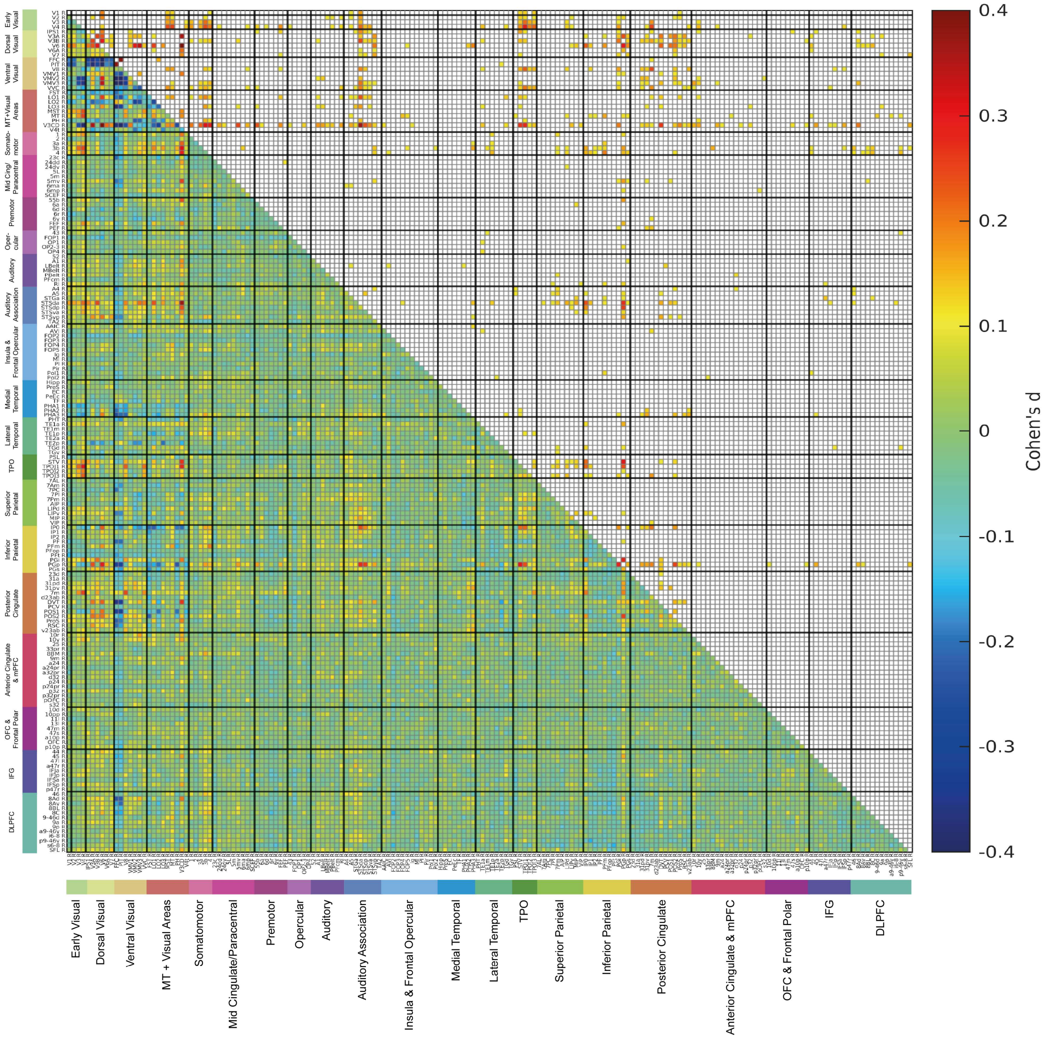


Fig. S4. The lower left triangle shows the matrix of functional connectivity differences between 0-back faces and the mean of all 0-back conditions processing in Working memory with the Cohen's d values showing the effect size of the differences. The matrix is for the functional connectivities in the *Right* hemisphere, as listed in Table S1, with V1, V2, V3 … at the top of the y axis and the left of the x axis. The upper triangle matrix shows the Cohen's d values of positively significant links after FDR correction (α=0.05). These results were from 956 participants in the HCP dataset. All the values shown in the matrix were limited to the range from -0.4 to 0.4. The covariates regressed out in this analysis were sex, age, drinker status, smoking status, education qualification and head motion.

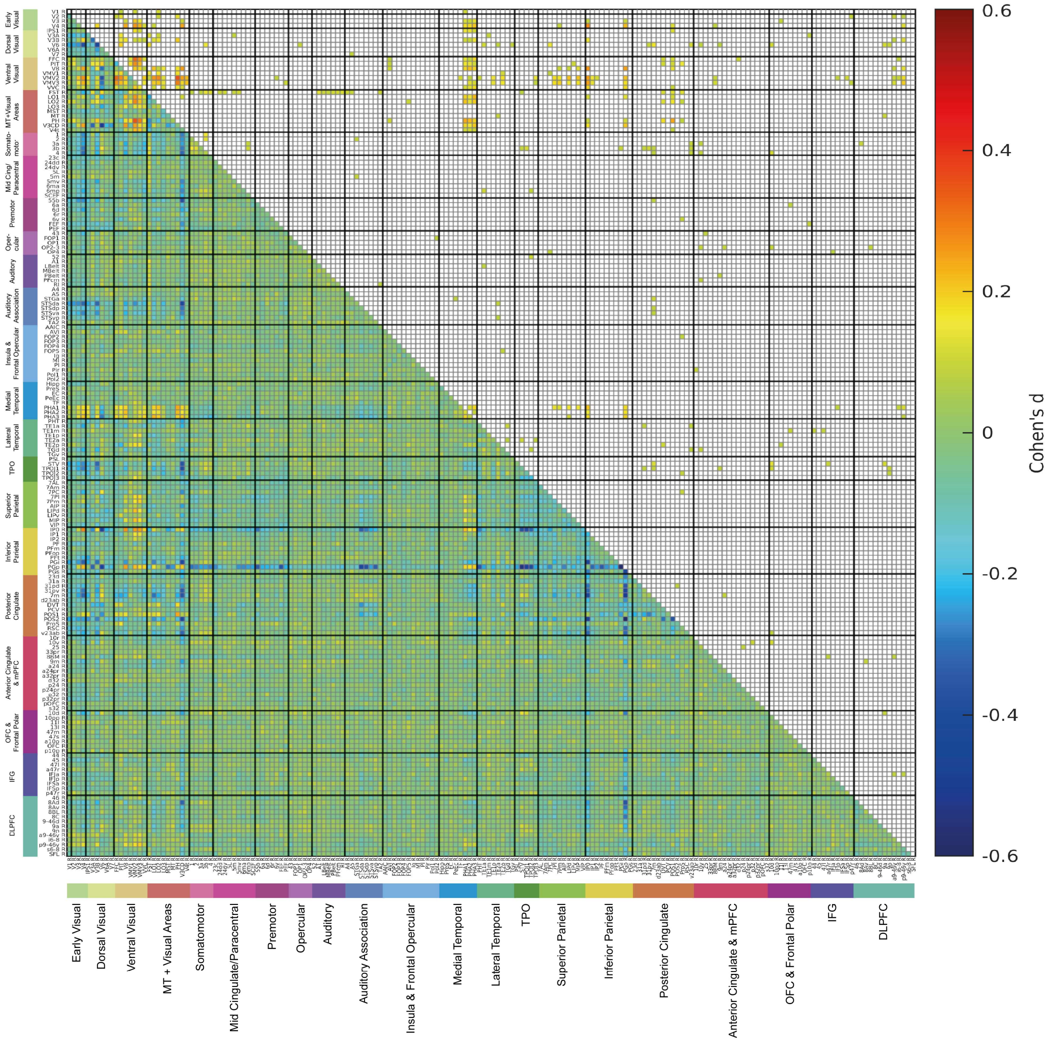


Fig. S5. The lower left triangle shows the matrix of functional connectivity differences between 0-back places (in fact scenes) and the mean of all 0-back conditions processing in Working memory with the Cohen's d values showing the effect size of the differences. The matrix is for the functional connectivities in the *Right* hemisphere, as listed in Table S1, with V1, V2, V3 … at the top of the y axis and the left of the x axis. The upper triangle matrix shows the Cohen's d values of positively significant links after FDR correction (α=0.05). These results were from 956 participants in the HCP dataset. All the values shown in the matrix were limited to the range from -0.6 to 0.6. The covariates regressed out in this analysis were sex, age, drinker status, smoking status, education qualification and head motion.

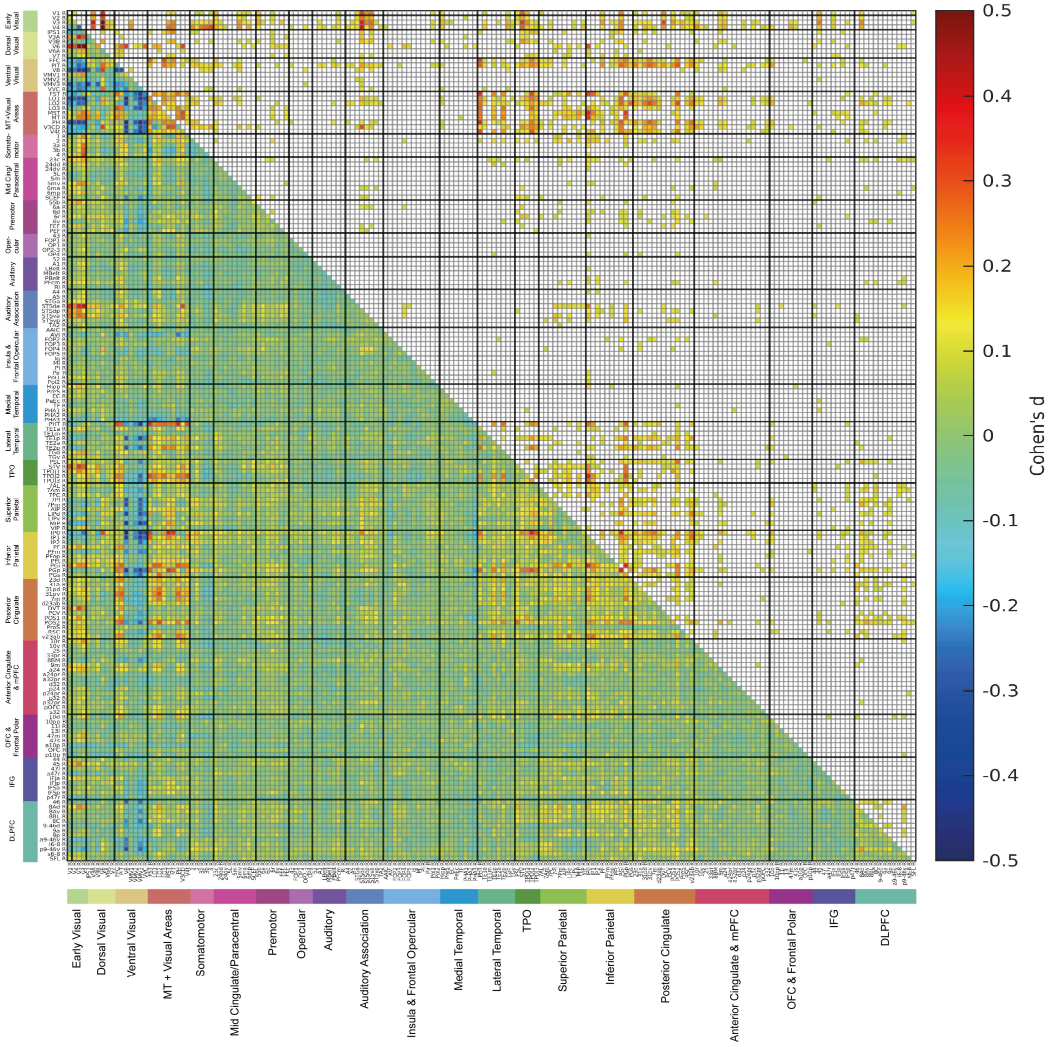


Fig. S6. The lower left triangle shows the matrix of functional connectivity differences between 0-back body parts and the mean of all 0-back conditions processing in Working memory with the Cohen's d values showing the effect size of the differences. The matrix is for the functional connectivities in the *Right* hemisphere, as listed in Table S1, with V1, V2, V3 … at the top of the y axis and the left of the x axis. The upper triangle matrix shows the Cohen's d values of positively significant links after FDR correction (α=0.05). These results were from 956 participants in the HCP dataset. All the values shown in the matrix were limited to the range from -0.5 to 0.5. The covariates regressed out in this analysis were sex, age, drinker status, smoking status, education qualification and head motion.

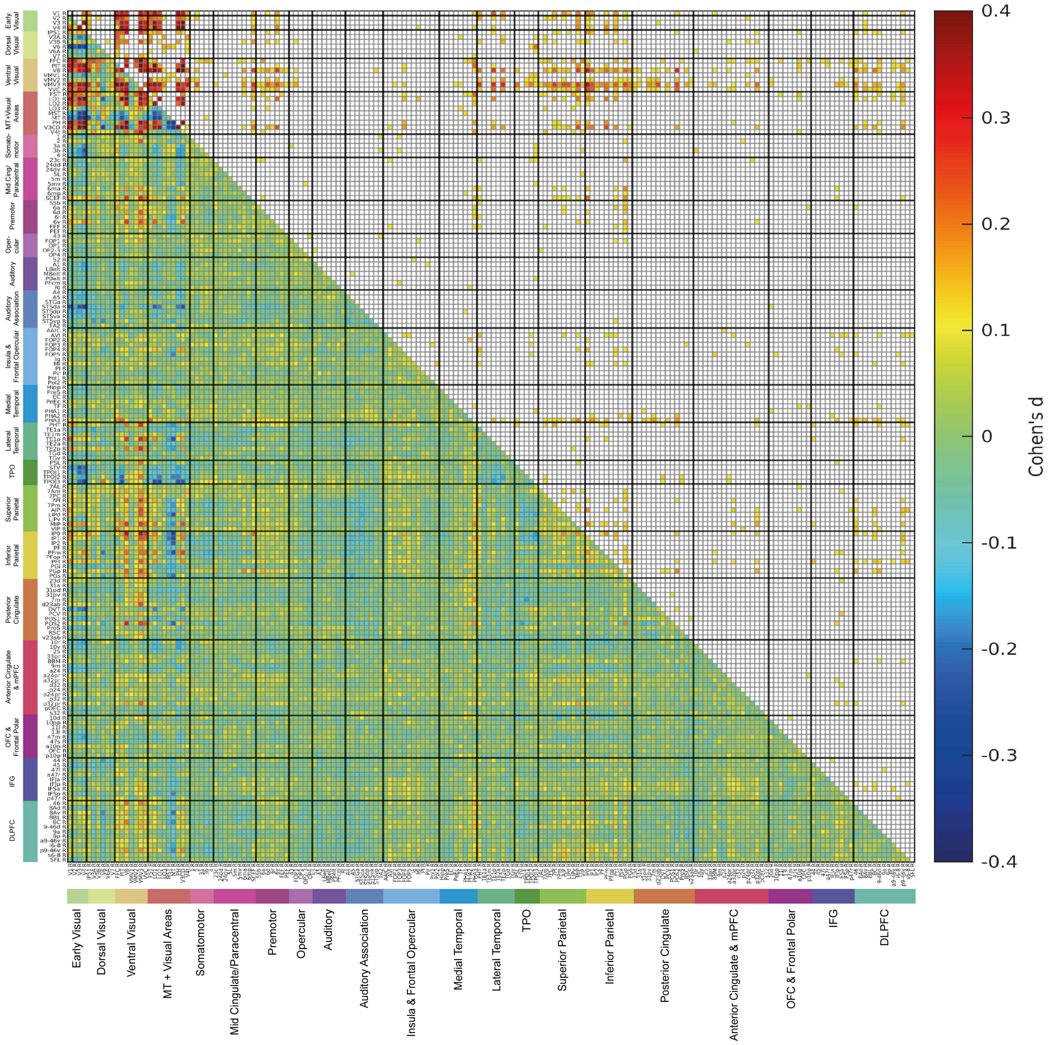


Fig. S7. The lower left triangle shows the matrix of functional connectivity differences between 0-back tools and the mean of all 0-back conditions processing in Working memory with the Cohen's d values showing the effect size of the differences. The matrix is for the functional connectivities in the *Right* hemisphere, as listed in Table S1, with V1, V2, V3 … at the top of the y axis and the left of the x axis. The upper triangle matrix shows the Cohen's d values of positively significant links after FDR correction (α=0.05). These results were from 956 participants in the HCP dataset. All the values shown in the matrix were limited to the range from -0.4 to 0.4. The covariates regressed out in this analysis were sex, age, drinker status, smoking status, education qualification and head motion.

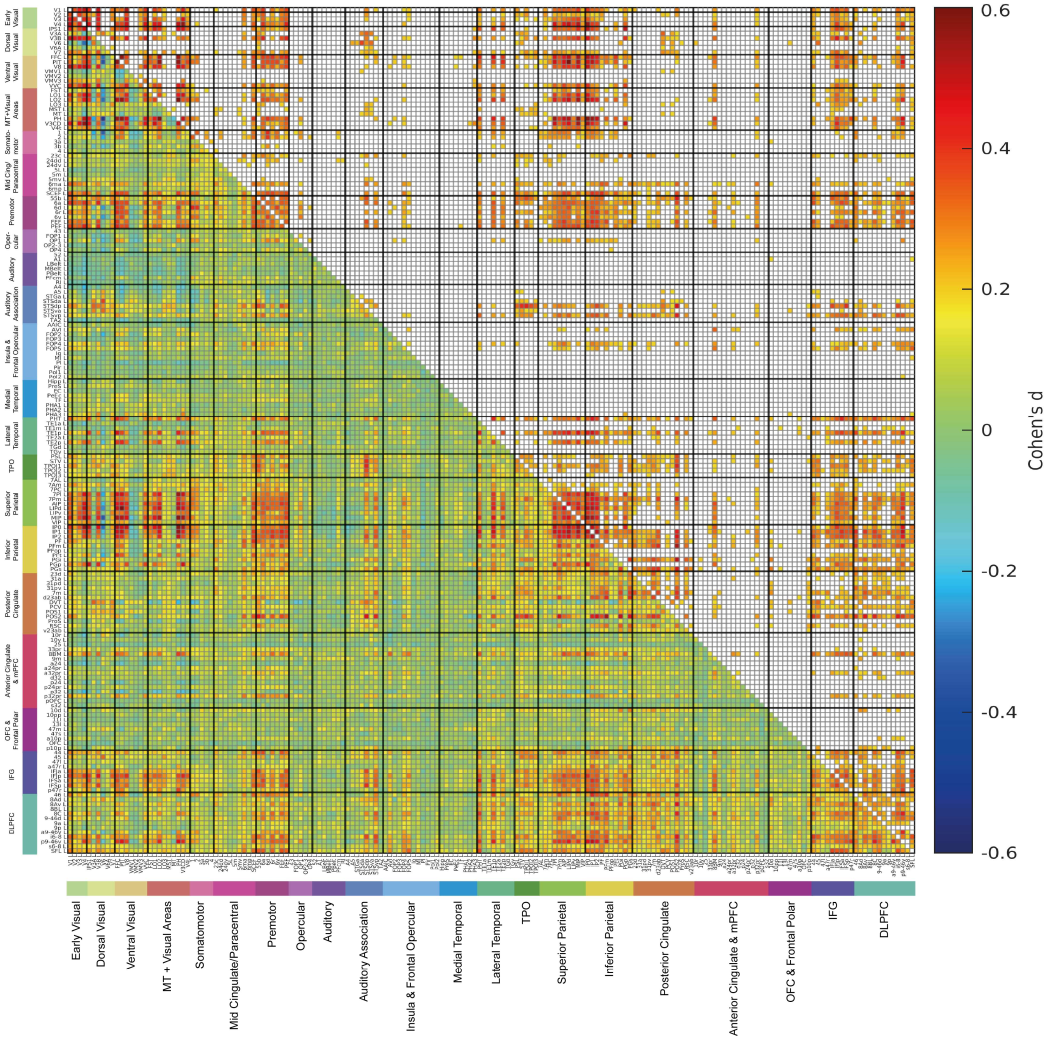


Fig. S8 The lower left triangle shows the matrix of functional connectivity differences between the initial 15 timepoints and the last 20 timepoints (when the BOLD signal was responding to the visual stimuli) of the mean across four 0-back working memory conditions and with the Cohen's d values showing the effect size of the differences. The matrix is for the functional connectivities in the *Left* hemisphere, as listed in Table S1, with V1, V2, V3 … at the top of the y axis and the left of the x axis. The upper triangle matrix shows the Cohen's d values of positively significant links after Bonferroni correction (α=0.001). These results were from 956 participants in the HCP dataset. All the values shown in the matrix were limited to the range from -0.6 to 0.6. The covariates regressed out in this analysis were sex, age, drinker status, smoking status, education qualification and head motion.

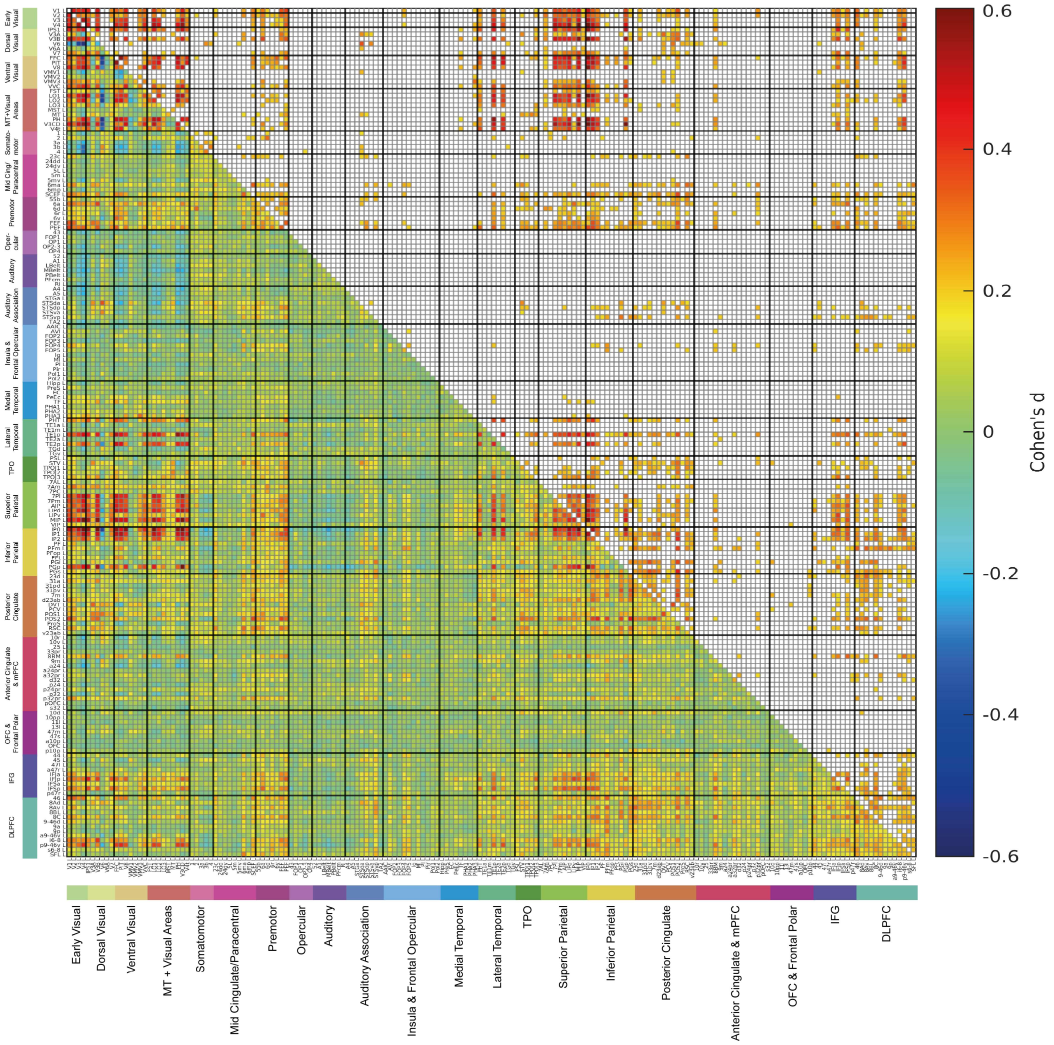


Fig. S9 The lower left triangle shows the matrix of functional connectivity differences between the initial 15 timepoints and the last 20 timepoints (when the BOLD signal was responding to the visual stimuli) of the mean across four 0-back working memory conditions with the Cohen's d values showing the effect size of the differences. The matrix is for the functional connectivities in the *Right* hemisphere, as listed in Table S1, with V1, V2, V3 … at the top of the y axis and the left of the x axis. The upper triangle matrix shows the Cohen's d values of positively significant links after Bonferroni correction (α=0.001). These results were from 956 participants in the HCP dataset. All the values shown in the matrix were limited to the range from -0.6 to 0.6. The covariates regressed out in this analysis were sex, age, drinker status, smoking status, education qualification and head motion.

**Table S2 The top 20 significant regions in the left hemisphere that were more positively activated by each of the four stimulus types (faces, paces, body parts, and tools) contrasted with the mean of the four stimulus types after Bonferroni correction (α=0.05) in the 0-back condition.** All p values are significant after Bonferroni correction apart from those shown in red font. The covariates stated in the text were removed from these analyses. These conventions apply to all the following Tables.

| **0-back faces vs. mean of the four 0-back stimuli** | | | **0-back places vs. mean of the four 0-back stimuli** | | |
|---|---|---|---|---|---|
| **Region** | **Cohen's d** | **p Value** | **Region** | **Cohen's d** | **p Value** |
| FFC L | 1.01 | $2.76\times10^{-147}$ | VMV2 L | 1.61 | $1.77\times10^{-268}$ |
| PGi L | 0.56 | $1.54\times10^{-58}$ | V3CD L | 1.35 | $1.21\times10^{-217}$ |
| TPOJ1 L | 0.50 | $9.84\times10^{-49}$ | VMV3 L | 1.34 | $6.16\times10^{-215}$ |
| PF L | 0.46 | $9.56\times10^{-42}$ | PGp L | 1.11 | $3.86\times10^{-169}$ |
| 7m L | 0.46 | $2.07\times10^{-41}$ | PHA3 L | 1.11 | $1.26\times10^{-168}$ |
| STV L | 0.45 | $5.71\times10^{-40}$ | V3B L | 1.04 | $1.71\times10^{-153}$ |
| PIT L | 0.44 | $1.91\times10^{-38}$ | PHA1 L | 1.03 | $5.28\times10^{-152}$ |
| TPOJ2 L | 0.43 | $6.63\times10^{-37}$ | PHA2 L | 0.94 | $6.30\times10^{-134}$ |
| OP1 L | 0.40 | $2.71\times10^{-32}$ | DVT L | 0.83 | $1.03\times10^{-111}$ |
| PFcm L | 0.38 | $3.13\times10^{-29}$ | V4 L | 0.69 | $9.57\times10^{-84}$ |
| PFop L | 0.37 | $6.03\times10^{-28}$ | IP0 L | 0.63 | $2.37\times10^{-72}$ |
| 6d L | 0.35 | $1.64\times10^{-25}$ | V3 L | 0.62 | $3.16\times10^{-69}$ |
| 55b L | 0.34 | $2.43\times10^{-24}$ | VMV1 L | 0.57 | $3.55\times10^{-61}$ |
| 2 L | 0.34 | $2.53\times10^{-24}$ | V8 L | 0.56 | $1.29\times10^{-58}$ |
| PSL L | 0.34 | $4.44\times10^{-24}$ | POS1 L | 0.49 | $1.96\times10^{-47}$ |
| 6ma L | 0.33 | $7.95\times10^{-23}$ | V1 L | 0.40 | $3.21\times10^{-32}$ |
| 1 L | 0.33 | $1.12\times10^{-22}$ | V2 L | 0.37 | $7.60\times10^{-29}$ |
| 31pd L | 0.32 | $6.91\times10^{-22}$ | ProS L | 0.35 | $2.89\times10^{-26}$ |
| 9p L | 0.31 | $6.92\times10^{-21}$ | 13l L | 0.30 | $4.20\times10^{-20}$ |
| RI L | 0.30 | $1.25\times10^{-19}$ | s32 L | 0.25 | $1.18\times10^{-14}$ |
| | | | | | |
| **0-back body parts vs. mean of the four 0-back stimuli** | | | **0-back tools vs. mean of the four 0-back stimuli** | | |
| **Region** | **Cohen's d** | **p Value** | **Region** | **Cohen's d** | **p Value** |
| V4t L | 1.23 | $4.70\times10^{-194}$ | VMV3 L | 0.91 | $4.25\times10^{-128}$ |
| FST L | 1.07 | $3.70\times10^{-160}$ | V1 L | 0.79 | $1.98\times10^{-103}$ |
| TPOJ3 L | 0.94 | $1.41\times10^{-132}$ | LO1 L | 0.76 | $4.15\times10^{-97}$ |
| PH L | 0.93 | $1.80\times10^{-131}$ | V3CD L | 0.73 | $2.01\times10^{-90}$ |

| | | | | | |
|---|---|---|---|---|---|
| PHT L | 0.92 | $3.37\times10^{-130}$ | V4 L | 0.72 | $5.07\times10^{-88}$ |
| TPOJ2 L | 0.85 | $2.39\times10^{-114}$ | PH L | 0.69 | $1.52\times10^{-82}$ |
| MT L | 0.84 | $5.29\times10^{-113}$ | FST L | 0.59 | $1.53\times10^{-64}$ |
| LO3 L | 0.83 | $3.00\times10^{-110}$ | V2 L | 0.58 | $7.91\times10^{-63}$ |
| FFC L | 0.79 | $6.14\times10^{-103}$ | VVC L | 0.53 | $1.04\times10^{-53}$ |
| POS2 L | 0.76 | $7.64\times10^{-97}$ | 13l L | 0.52 | $3.91\times10^{-51}$ |
| LO2 L | 0.76 | $6.46\times10^{-96}$ | V3 L | 0.50 | $1.10\times10^{-48}$ |
| IP1 L | 0.64 | $4.40\times10^{-73}$ | V3A L | 0.48 | $1.44\times10^{-44}$ |
| IP2 L | 0.61 | $2.69\times10^{-67}$ | PHA3 L | 0.42 | $5.94\times10^{-35}$ |
| AIP L | 0.60 | $2.56\times10^{-65}$ | V8 L | 0.39 | $1.32\times10^{-31}$ |
| 8BM L | 0.56 | $1.68\times10^{-58}$ | pOFC L | 0.36 | $1.06\times10^{-27}$ |
| PGi L | 0.53 | $9.69\times10^{-54}$ | V3B L | 0.35 | $1.80\times10^{-26}$ |
| MST L | 0.52 | $8.19\times10^{-52}$ | OFC L | 0.32 | $6.72\times10^{-22}$ |
| TE2p L | 0.52 | $9.01\times10^{-52}$ | V6 L | 0.29 | $4.32\times10^{-19}$ |
| PGs L | 0.49 | $2.02\times10^{-47}$ | IPS1 L | 0.27 | $9.82\times10^{-17}$ |
| 47l L | 0.49 | $1.76\times10^{-46}$ | LO2 L | 0.26 | $1.21\times10^{-15}$ |

**Table S3 The activations of the memory-related regions in the left hemisphere for each of the four stimuli faces, places, body parts, and tools contrasted with the mean of the four stimuli, in the 0-back conditions**

| 0-back faces vs. mean of the four 0-back stimuli | | | 0-back places vs. mean of the four 0-back stimuli | | |
|---|---|---|---|---|---|
| Region | Cohen's d | p Value | Region | Cohen's d | p Value |
| VMV1 L | -0.48 | $1.56\times10^{-45}$ | VMV1 L | 0.57 | $3.55\times10^{-61}$ |
| VMV2 L | -1.13 | $1.22\times10^{-173}$ | VMV2 L | 1.61 | $1.77\times10^{-268}$ |
| VMV3 L | -1.37 | $1.13\times10^{-220}$ | VMV3 L | 1.34 | $6.16\times10^{-215}$ |
| VVC L | -0.59 | $1.01\times10^{-64}$ | VVC L | 0.21 | $2.28\times10^{-10}$ |
| Hipp L | 0.08 | 0.01 | Hipp L | -0.31 | $2.19\times10^{-21}$ |
| PreS L | -0.04 | 0.17 | PreS L | -0.02 | 0.51 |
| EC L | -0.13 | $1.13\times10^{-4}$ | EC L | -0.31 | $9.39\times10^{-21}$ |
| PeEc L | -0.14 | $1.32\times10^{-5}$ | PeEc L | -0.51 | $3.13\times10^{-50}$ |
| TF L | 0.30 | $1.99\times10^{-19}$ | TF L | -0.42 | $3.08\times10^{-36}$ |
| PHA1 L | -0.48 | $6.91\times10^{-45}$ | PHA1 L | 1.03 | $5.28\times10^{-152}$ |
| PHA2 L | -0.72 | $2.97\times10^{-88}$ | PHA2 L | 0.94 | $6.30\times10^{-134}$ |
| PHA3 L | -1.24 | $6.69\times10^{-195}$ | PHA3 L | 1.11 | $1.26\times10^{-168}$ |
| | | | | | |
| **0-back body parts vs. mean of the four 0-back stimuli** | | | **0-back tools vs. mean of the four 0-back stimuli** | | |
| **Region** | **Cohen's d** | **p Value** | **Region** | **Cohen's d** | **p Value** |
| VMV1 L | -0.04 | 0.21 | VMV1 L | -0.01 | 0.79 |
| VMV2 L | -0.42 | $2.56\times10^{-36}$ | VMV2 L | 0.12 | $3.52\times10^{-4}$ |
| VMV3 L | -0.75 | $6.62\times10^{-94}$ | VMV3 L | 0.91 | $4.25\times10^{-128}$ |
| VVC L | -0.13 | $8.23\times10^{-5}$ | VVC L | 0.53 | $1.04\times10^{-53}$ |
| Hipp L | 0.29 | $4.47\times10^{-19}$ | Hipp L | -0.12 | $1.78\times10^{-4}$ |
| PreS L | 0.24 | $1.02\times10^{-13}$ | PreS L | -0.18 | $3.99\times10^{-8}$ |
| EC L | 0.38 | $1.99\times10^{-30}$ | EC L | 0.01 | 0.71 |
| PeEc L | 0.45 | $9.21\times10^{-41}$ | PeEc L | 0.11 | $5.28\times10^{-4}$ |
| TF L | 0.25 | $1.56\times10^{-14}$ | TF L | -0.18 | $6.42\times10^{-8}$ |
| PHA1 L | -0.10 | $1.60\times10^{-3}$ | PHA1 L | -0.39 | $5.58\times10^{-31}$ |
| PHA2 L | -0.12 | $2.78\times10^{-4}$ | PHA2 L | -0.11 | $6.19\times10^{-4}$ |
| PHA3 L | -0.26 | $1.59\times10^{-15}$ | PHA3 L | 0.42 | $5.94\times10^{-35}$ |

**Table S4. The top 20 significant regions in the right hemisphere that were more positively activated by each of the four stimulus types (faces, paces, body parts, and tools) contrasted with the mean of the four stimulus types after Bonferroni correction (α=0.05) in the 0-back condition.**

| **0-back faces vs. mean of the four 0-back stimuli** | | | **0-back places vs. mean of the four 0-back stimuli** | | |
|---|---|---|---|---|---|
| **Region** | **Cohen's d** | **p Value** | **Region** | **Cohen's d** | **p Value** |
| FFC R | 1.28 | $4.87\times10^{-204}$ | VMV2 R | 2.04 | $<1.0\times10^{-15}$ |
| PIT R | 0.72 | $3.41\times10^{-88}$ | V3CD R | 1.91 | $<1.0\times10^{-15}$ |
| 7m R | 0.61 | $1.74\times10^{-67}$ | VMV3 R | 1.77 | $5.57\times10^{-296}$ |
| TPOJ1 R | 0.58 | $2.01\times10^{-61}$ | PHA3 R | 1.70 | $1.01\times10^{-284}$ |
| STSda R | 0.50 | $1.51\times10^{-48}$ | PHA2 R | 1.52 | $1.08\times10^{-250}$ |
| pOFC R | 0.49 | $3.88\times10^{-46}$ | DVT R | 1.44 | $1.22\times10^{-234}$ |
| SCEF R | 0.48 | $8.46\times10^{-46}$ | V3B R | 1.41 | $3.99\times10^{-229}$ |
| STSdp R | 0.48 | $5.58\times10^{-45}$ | PGp R | 1.41 | $5.79\times10^{-229}$ |
| TGd R | 0.44 | $3.66\times10^{-39}$ | PHA1 R | 1.35 | $2.14\times10^{-217}$ |
| 24dd R | 0.43 | $1.53\times10^{-37}$ | IP0 R | 1.18 | $3.39\times10^{-184}$ |
| 24dv R | 0.43 | $2.06\times10^{-36}$ | V4 R | 1.06 | $1.65\times10^{-158}$ |
| 13l R | 0.42 | $6.02\times10^{-35}$ | POS1 R | 1.05 | $3.33\times10^{-156}$ |
| STSva R | 0.40 | $2.54\times10^{-32}$ | V3 R | 1.01 | $3.53\times10^{-148}$ |
| 55b R | 0.39 | $6.79\times10^{-31}$ | V8 R | 0.95 | $7.12\times10^{-136}$ |
| TPOJ2 R | 0.38 | $5.28\times10^{-30}$ | V2 R | 0.88 | $8.47\times10^{-121}$ |
| p24pr R | 0.35 | $4.85\times10^{-26}$ | V1 R | 0.80 | $4.42\times10^{-104}$ |
| PoI1 R | 0.34 | $5.65\times10^{-25}$ | VVC R | 0.75 | $1.44\times10^{-95}$ |
| p32pr R | 0.33 | $6.76\times10^{-24}$ | VMV1 R | 0.72 | $1.18\times10^{-89}$ |
| 6d R | 0.32 | $2.61\times10^{-22}$ | ProS R | 0.65 | $9.95\times10^{-76}$ |
| 52 R | 0.31 | $6.15\times10^{-21}$ | V3A R | 0.65 | $8.72\times10^{-75}$ |
| | | | | | |
| **0-back body parts vs. mean of the four 0-back stimuli** | | | **0-back tools vs. mean of the four 0-back stimuli** | | |
| **Region** | **Cohen's d** | **p Value** | **Region** | **Cohen's d** | **p Value** |
| FST R | 1.08 | $4.47\times10^{-163}$ | V1 R | 0.86 | $4.17\times10^{-117}$ |
| V4t R | 0.97 | $9.71\times10^{-140}$ | VMV3 R | 0.82 | $1.09\times10^{-109}$ |
| PH R | 0.96 | $1.43\times10^{-137}$ | LO1 R | 0.61 | $8.31\times10^{-68}$ |
| LO3 R | 0.75 | $3.16\times10^{-94}$ | V3CD R | 0.59 | $7.00\times10^{-65}$ |
| MT R | 0.69 | $2.83\times10^{-82}$ | V2 R | 0.59 | $3.60\times10^{-64}$ |
| TPOJ3 R | 0.66 | $7.72\times10^{-78}$ | V4 R | 0.58 | $2.22\times10^{-61}$ |

| | | | | | |
|---|---|---|---|---|---|
| PHT R | 0.62 | $6.30\times10^{-70}$ | V3A R | 0.56 | $5.39\times10^{-59}$ |
| POS2 R | 0.62 | $4.81\times10^{-69}$ | VVC R | 0.52 | $3.93\times10^{-52}$ |
| LO2 R | 0.60 | $4.05\times10^{-66}$ | V3 R | 0.50 | $3.75\times10^{-48}$ |
| TE2p R | 0.60 | $2.97\times10^{-65}$ | V8 R | 0.49 | $7.84\times10^{-46}$ |
| TPOJ2 R | 0.53 | $5.07\times10^{-54}$ | V6 R | 0.41 | $2.63\times10^{-34}$ |
| FFC R | 0.53 | $4.47\times10^{-53}$ | PH R | 0.37 | $1.40\times10^{-28}$ |
| MST R | 0.44 | $7.99\times10^{-39}$ | IPS1 R | 0.32 | $1.74\times10^{-22}$ |
| AIP R | 0.44 | $1.65\times10^{-38}$ | V7 R | 0.30 | $5.11\times10^{-20}$ |
| MIP R | 0.42 | $2.08\times10^{-35}$ | FST R | 0.24 | $2.69\times10^{-13}$ |
| 8BM R | 0.40 | $4.67\times10^{-33}$ | VMV2 R | 0.22 | $8.13\times10^{-12}$ |
| IFSa R | 0.38 | $1.81\times10^{-29}$ | LIPv R | 0.21 | $2.00\times10^{-10}$ |
| LO1 R | 0.36 | $3.30\times10^{-27}$ | POS2 R | 0.20 | $1.75\times10^{-9}$ |
| IPS1 R | 0.35 | $3.08\times10^{-25}$ | 1 R | 0.18 | $2.68\times10^{-8}$ |
| 8BL R | 0.34 | $4.20\times10^{-25}$ | PHA3 R | 0.17 | $9.66\times10^{-8}$ |

**Table S5 The activations of the memory-related regions in the right hemisphere for each of the four stimuli faces, places, body parts, and tools contrasted with the mean of the four stimuli, in the 0-back conditions.**

| **0-back faces vs. mean of the four 0-back stimuli** | | | **0-back places vs. mean of the four 0-back stimuli** | | |
|---|---|---|---|---|---|
| **Region** | **Cohen's d** | **p Value** | **Region** | **Cohen's d** | **p Value** |
| VMV1 R | -0.63 | $5.16\times10^{-71}$ | VMV1 R | 0.72 | $1.18\times10^{-89}$ |
| VMV2 R | -1.52 | $9.40\times10^{-250}$ | VMV2 R | 2.04 | $<1.0\times10^{-15}$ |
| VMV3 R | -1.61 | $1.05\times10^{-267}$ | VMV3 R | 1.77 | $5.57\times10^{-296}$ |
| VVC R | -0.86 | $3.75\times10^{-116}$ | VVC R | 0.75 | $1.44\times10^{-95}$ |
| Hipp R | 0.25 | $2.30\times10^{-14}$ | Hipp R | -0.04 | 0.19 |
| PreS R | -0.04 | 0.23 | PreS R | 0.20 | $1.79\times10^{-9}$ |
| EC R | 0.18 | $2.57\times10^{-8}$ | EC R | -0.04 | 0.28 |
| PeEc R | 0.28 | $1.58\times10^{-17}$ | PeEc R | -0.15 | $4.33\times10^{-6}$ |
| TF R | 0.05 | 0.11 | TF R | -0.06 | 0.07 |
| PHA1 R | -0.64 | $1.97\times10^{-73}$ | PHA1 R | 1.35 | $2.14\times10^{-217}$ |
| PHA2 R | -0.91 | $4.91\times10^{-127}$ | PHA2 R | 1.52 | $1.08\times10^{-250}$ |
| PHA3 R | -1.25 | $2.56\times10^{-198}$ | PHA3 R | 1.70 | $1.01\times10^{-284}$ |
| | | | | | |
| **0-back body parts vs. mean of the four 0-back stimuli** | | | **0-back tools vs. mean of the four 0-back stimuli** | | |
| **Region** | **Cohen's d** | **p Value** | **Region** | **Cohen's d** | **p Value** |
| VMV1 R | -0.16 | $1.09\times10^{-6}$ | VMV1 R | 0.09 | 0.01 |
| VMV2 R | -0.73 | $1.92\times10^{-91}$ | VMV2 R | 0.22 | $8.13\times10^{-12}$ |
| VMV3 R | -0.89 | $1.61\times10^{-122}$ | VMV3 R | 0.82 | $1.09\times10^{-109}$ |
| VVC R | -0.31 | $8.48\times10^{-21}$ | VVC R | 0.52 | $3.93\times10^{-52}$ |
| Hipp R | -0.05 | 0.11 | Hipp R | -0.16 | $7.39\times10^{-7}$ |
| PreS R | 0.06 | 0.05 | PreS R | -0.22 | $9.69\times10^{-12}$ |
| EC R | 0.05 | 0.15 | EC R | -0.19 | $1.22\times10^{-8}$ |
| PeEc R | -0.01 | 0.76 | PeEc R | -0.13 | $4.05\times10^{-5}$ |
| TF R | 0.06 | 0.06 | TF R | -0.06 | 0.06 |
| PHA1 R | -0.34 | $3.93\times10^{-24}$ | PHA1 R | -0.41 | $2.25\times10^{-34}$ |
| PHA2 R | -0.46 | $9.54\times10^{-42}$ | PHA2 R | -0.24 | $5.19\times10^{-13}$ |
| PHA3 R | -0.54 | $8.10\times10^{-56}$ | PHA3 R | 0.17 | $9.66\times10^{-8}$ |

**Table S6 The top 20 significant regions in the left hemisphere that were more positively activated by the visual stimuli in the 2-back contrasted with the 0-back task after the Bonferroni correction (α=0.05)**

| 2-back faces vs 0-back faces | | | 2-back places vs 0-back places | | |
|---|---|---|---|---|---|
| **Region** | **Cohen's d** | **p Value** | **Region** | **Cohen's d** | **p Value** |
| 7Pm L | 0.78 | $2.38\times10^{-100}$ | POS2 L | 0.81 | $3.35\times10^{-106}$ |
| i6-8 L | 0.61 | $7.60\times10^{-68}$ | PFm L | 0.79 | $5.83\times10^{-103}$ |
| 13l L | 0.57 | $1.40\times10^{-59}$ | 7Pm L | 0.78 | $5.64\times10^{-100}$ |
| IP2 L | 0.56 | $5.60\times10^{-58}$ | 7m L | 0.77 | $6.65\times10^{-98}$ |
| p9-46v L | 0.50 | $1.53\times10^{-47}$ | IP2 L | 0.71 | $2.57\times10^{-87}$ |
| IP1 L | 0.46 | $2.83\times10^{-42}$ | i6-8 L | 0.67 | $9.41\times10^{-80}$ |
| OFC L | 0.46 | $3.52\times10^{-42}$ | PGs L | 0.63 | $1.55\times10^{-71}$ |
| 8BM L | 0.45 | $1.84\times10^{-39}$ | IP1 L | 0.55 | $6.24\times10^{-57}$ |
| pOFC L | 0.43 | $2.83\times10^{-37}$ | 31pd L | 0.48 | $1.57\times10^{-44}$ |
| 7Pl L | 0.41 | $9.36\times10^{-34}$ | 8Av L | 0.46 | $1.53\times10^{-42}$ |
| a9-46v L | 0.39 | $1.82\times10^{-31}$ | a9-46v L | 0.46 | $1.24\times10^{-41}$ |
| 11l L | 0.36 | $4.93\times10^{-27}$ | PGi L | 0.45 | $8.42\times10^{-41}$ |
| 8C L | 0.36 | $1.20\times10^{-26}$ | 31pv L | 0.45 | $1.22\times10^{-40}$ |
| AVI L | 0.35 | $1.84\times10^{-25}$ | p9-46v L | 0.43 | $7.68\times10^{-38}$ |
| PFm L | 0.34 | $3.95\times10^{-25}$ | 8C L | 0.43 | $3.45\times10^{-37}$ |
| SFL L | 0.34 | $6.43\times10^{-24}$ | s6-8 L | 0.41 | $1.62\times10^{-34}$ |
| LIPd L | 0.30 | $5.63\times10^{-20}$ | 8BM L | 0.41 | $8.32\times10^{-34}$ |
| POS2 L | 0.30 | $1.42\times10^{-19}$ | RSC L | 0.40 | $4.83\times10^{-33}$ |
| p10p L | 0.28 | $1.01\times10^{-17}$ | SFL L | 0.38 | $8.52\times10^{-30}$ |
| a10p L | 0.27 | $9.94\times10^{-17}$ | 6ma L | 0.37 | $6.63\times10^{-28}$ |
| | | | | | |
| **2-back body parts vs 0-back body parts** | | | **2-back tools vs 0-back tools** | | |
| **Region** | **Cohen's d** | **p Value** | **Region** | **Cohen's d** | **p Value** |
| AIP L | 0.49 | $2.26\times10^{-46}$ | IP2 L | 0.73 | $5.62\times10^{-90}$ |
| 7Pm L | 0.46 | $5.22\times10^{-42}$ | AIP L | 0.70 | $2.57\times10^{-84}$ |
| IP2 L | 0.40 | $2.03\times10$-$^{33}$ | 7Pm L | 0.68 | $4.78\times10^{-81}$ |
| 6a L | 0.40 | $5.15\times10^{-33}$ | 6a L | 0.65 | $2.00\times10^{-75}$ |
| 6ma L | 0.39 | $1.50\times10^{-31}$ | i6-8 L | 0.65 | $8.80\times10^{-75}$ |
| PFt L | 0.34 | $2.00\times10^{-24}$ | SCEF L | 0.64 | $4.14\times10^{-73}$ |
| PFm L | 0.33 | $4.34\times10^{-23}$ | 6ma L | 0.62 | $6.74\times10^{-70}$ |
| V4 L | 0.33 | $5.83\times10^{-23}$ | LIPd L | 0.60 | $6.03\times10^{-66}$ |
| 7Pl L | 0.32 | $7.56\times10^{-22}$ | PFm L | 0.56 | $7.80\times10^{-59}$ |

| | | | | | |
|---|---|---|---|---|---|
| 55b L | 0.31 | $1.47\times10^{-20}$ | p32pr L | 0.54 | $1.51\times10^{-54}$ |
| i6-8 L | 0.31 | $2.48\times10^{-20}$ | 7Pl L | 0.54 | $1.57\times10^{-54}$ |
| V8 L | 0.28 | $2.75\times10^{-17}$ | 8BM L | 0.50 | $1.52\times10^{-48}$ |
| FEF L | 0.28 | $6.68\times10^{-17}$ | FEF L | 0.50 | $2.00\times10^{-48}$ |
| s6-8 L | 0.27 | $1.87\times10^{-16}$ | SFL L | 0.50 | $1.28\times10^{-47}$ |
| SCEF L | 0.26 | $2.08\times10^{-15}$ | a32pr L | 0.49 | $2.61\times10^{-47}$ |
| p9-46v L | 0.25 | $2.21\times10^{-14}$ | 55b L | 0.48 | $8.83\times10^{-45}$ |
| LIPd L | 0.24 | $3.96\times10^{-13}$ | p9-46v L | 0.46 | $3.59\times10^{-41}$ |
| 6r L | 0.23 | $3.89\times10^{-12}$ | s6-8 L | 0.45 | $1.54\times10^{-40}$ |
| PEF L | 0.23 | $4.75\times10^{-12}$ | IP1 L | 0.44 | $5.55\times10^{-38}$ |
| 6d L | 0.22 | $8.46\times10^{-12}$ | a9-46v L | 0.42 | $2.76\times10^{-36}$ |

References

Glasser MF, Coalson TS, Robinson EC, Hacker CD, Harwell J, Yacoub E, Ugurbil K, Andersson J, Beckmann CF, Jenkinson M, Smith SM, Van Essen DC (2016) A multi-modal parcellation of human cerebral cortex. Nature 536 (7615):171-178. doi:10.1038/nature18933

Huang C-C, Rolls ET, Feng J, Lin C-P (2022) An extended Human Connectome Project multimodal parcellation atlas of the human cortex and subcortical areas. Brain Structure and Function 227 (3):763-778
